# Complementary approaches define the metabolic features that accompany Richter syndrome transformation

**DOI:** 10.1007/s00018-025-05670-4

**Published:** 2025-04-09

**Authors:** Giulia Omezzolli, Andrea Iannello, Francesco E. Vallone, Lorenzo Brandimarte, Matilde Micillo, Nadia Bertola, Chiara Lavarello, Nicole Grinovero, Giulio Ferrero, Kevin Mellert, Peter Möller, Silvia Bruno, Richard R. Furman, John N. Allan, Andrea Petretto, Silvia Deaglio, Silvia Ravera, Tiziana Vaisitti

**Affiliations:** 1 Department of Medical Sciences, University of Torino, Via Nizza 52, 10126 Turin, Italy; 2 U.O. Molecular Pathology, IRCCS Ospedale Policlinico San Martino, Genoa, Italy; 3 Core Facilities-Clinical Proteomics and Metabolomics, IRCCS Giannina Gaslini, Genoa, Italy; 4 Department of Clinical and Biological Sciences, University of Turin, Turin, Italy; 5 Institute of Pathology, University Hospital Ulm, Ulm, Germany; 6 Department of Experimental Medicine, University of Genoa, Genoa, Italy; 7 Weill Cornell Medicine, NewYork-Presbyterian Hospital, New York, NY USA; 8 IRCCS Ospedale Policlinico San Martino, Genova, Italy

**Keywords:** Richter transformation, Chronic lymphocytic leukemia, Metabolic rewiring, Metabolic dependencies, Building blocks, Metabolic targeting

## Abstract

Richter syndrome (RS) is the transformation of chronic lymphocytic leukemia (CLL) into a high-grade lymphoma with previously unknown metabolic features. Transcriptomic data from primary CLL and RS samples, as well as RS-patient-derived xenografts, highlighted cellular metabolism as one of the most significant differentially expressed processes. Activity assays of key enzymes confirmed the intense metabolic rewiring of RS cells, which is characterized by an elevated rate of Krebs cycle, oxidative phosphorylation, and glutamine metabolism. These pathways were sustained by increased uptake of glucose and glutamine, two critical substrates for these cells. Moreover, RS cells showed activation of anabolic processes that resulted in the synthesis of nucleotides and lipids necessary to support their high proliferation. Exposure to drugs targeting PI3K and NF-kB, two master regulators of cellular metabolism, resulted in the shutdown of ATP production and glycolysis. Overall, these data suggest that metabolic rewiring characterizes the transformation of CLL into RS, presenting new translational opportunities.

**Supplementary Information:**

The online version contains supplementary material available at 10.1007/s00018-025-05670-4.

## Introduction

Cellular metabolism reprogramming is a known hallmark of cancer [1]. Detrimental survival programs and a high proliferative capacity of tumor cells result in altered metabolic fluxes to meet increased bioenergetic and biosynthetic demands [2]. Most cancer cells show enhanced glucose uptake due to oncogenic driver mutations and environmental nutrient availability. The tumor microenvironment forces cancer cells to adapt their metabolism based on substrate availability and oxygen concentration [3]. Normally, glucose is processed to generate pyruvate, which enters the tricarboxylic acid (TCA) cycle and fuels oxidative phosphorylation (OXPHOS) to sustain the energy demand and restore nicotinamide adenine dinucleotide (NAD^+^) levels [4, 5].

Besides catabolic pathways, a portion of glucose is redirected into anabolic cascades to generate the nucleotides and phospholipids necessary for the de novo synthesis of macromolecules to sustain cancer cell growth [6, 7]. In this complex metabolic network, the TCA cycle is at the crossroads between catabolic and anabolic pathways since its intermediates are often the precursors for macromolecule synthesis [1].

However, while general statements about cancer metabolism can be made, it is well known that tumor cells can be heterogeneous in their metabolic demands and adaptations [2].

Richter syndrome (RS) is defined as the development of a high-grade aggressive lymphoma in a patient with a previous or concomitant diagnosis of chronic lymphocytic leukemia (CLL) [8]. In recent years, efforts have been made to define the metabolic features of CLL cells, highlighting differences from normal B cells and high metabolic plasticity because of leukemic cell recirculation from peripheral blood (PB) to lymphoid (LN) compartments. Jitschin and colleagues showed that quiescent PB CLL cells had higher OXPHOS, but not glycolysis, than healthy B cells together with an increased production of reactive oxygen species (ROS) fueled by the OXPHOS [9]. Moreover, CLL metabolism depends on lipids, stored in cytoplasmic vacuoles, which fuel beta-oxidation [10–12]. When in the LN, because of signals coming from the microenvironment (e.g., BCR, CD40) CLL cells become addicted to glutamine, a key substrate that feeds TCA, together with a shift of glucose towards lactate production and branching pathways [13, 14].

This study aims to provide a snapshot of the metabolic landscape of RS cells by utilizing 4 patient-derived tumor xenograft (RS-PDX) models [15, 16], and an RS cell line, U-RT1 [17]. We analyzed expression and activity of key enzymes involved in various metabolic pathways, comparing RS to CLL cells, to define the metabolic differences that characterize these lymphoma cells and allow them to sustain their higher proliferative behavior. Metabolic dependencies of RS were also identified, determining the substrates RS cells rely on to produce energy and the building blocks necessary to support their active proliferation. These results were further validated using a metabolomic approach. Understanding and characterizing the metabolic features of RS may open new translational perspectives, exploiting the RS metabolic fingerprint as a potential target.

## Methods

### RS-patient-derived xenograft models (RS-PDX)

RS-PDX models were established and maintained as described [15, 16]. The Institutional Animal Care and Use Committee approved all the experiments involving mice. Mice were treated following the European guidelines and with the approval of the Italian Ministry of Health (authorization #664/2020-PR).

### RNA sequencing and transcriptomic data analysis

RNA-Seq was performed as described [15, 16]. Briefly, RNA was extracted using Qiazol (Qiagen, Hilden, DE) and quantified using the Qubit RNA HS assay kit (Life Technologies, Carlsbad, USA), while RNA quality was assessed with the Bioanalyzer RNA 6000 Nano kit (Agilent, Santa Clara, USA). 500 ng of total RNA was subjected to the RNA-Seq library preparation using the TruSeq RNA Sample Prep Kit or the TruSeq stranded. Details on bioinformatic analyses are provided in Supplementary data.

### Glucose and glutamine uptake

RS-PDXs and CLL patients’ cells were starved overnight (O.N.) in DMEM low glucose 0.1% FBS, washed, incubated with 50 μM 2-(*N*-(7-Nitrobenz-2-oxa-1,3-diazol-4-yl)Amino)-2-Deoxyglucose (2-NBDG, Sigma Aldrich) for different time points (30, 15, 10, 5, 3 and 1 min) at 37 °C, washed and resuspended in a wash buffer for cytofluorimetry and confocal microscopy analyses. Negative controls were performed in the absence of 2-NBDG. For confocal microscopy image analyses, the 2-NBDG fluorescence mean intensity (MFI) for each cell was assessed with an ImageJ macro analysis, identifying cells in the bright field. The glutamine uptake protocol was performed using the Glutamine/Glutamate Determination Kit (Merck, Milan) following manufacturer’s instruction. The starting concentration of glutamine in the medium was measured in RPMI 10% FCS only, without cells. A standard curve was prepared using acetate buffer, glutaminase solution, and 2 mM Glutamine Standard diluted in ultrapure water at different increasing concentrations. Standard and test samples were processed with the Biotek instrument (absorbance 340 nm) and glutamine sample concentration obtained. Endogenous L-glutamate concentration was subtracted from total L-glutamate concentration, resulting in L-glutamate concentration arising from deamination of L-glutamine. The L-glutamine concentration after O.N. incubation was subtracted from the L-glutamine concentration at the basal situation. Finally, the Δ of L-glutamine in RPMI was subtracted from the Δ of each sample, determining the L-glutamine uptake value.

### Seahorse experiments

RS cells were seeded in RPMI medium supplemented with fetal calf serum (FCS) and treated with either the dual PI3K γ/δ inhibitor duvelisib (DUV) or the NF-κB inhibitor (sc75741), both from Selleckchem (Aurogene, Rome) for 24 h. For the Mitostress test (Agilent Technologies Inc., Wilmington, DE, United States), cells were harvested and suspended in XF assay medium containing 10 mM Glucose, 1 mM Pyruvate, and 2 mM L-Glutamine. A seeding density of 180,000 cells per well (U-RT1) or 230,000 cells per well (RS-PDXs) was employed in XF24 cell culture microplates, precoated with poly-lysine (Agilent Technologies Inc.). The assay was performed following manufacturer’s instructions using oligomycin, FCCP and rotenone/antimycin A (1.5 μM, 0.25 µM and 0.5 µM, respectively). For the Glycostress test (Agilent Technologies Inc.) RS cells were resuspended in XF assay medium containing 2 mM L-Glutamine with serial injections of Glucose, Oligomycin, and 2-DG (10 mM, 1 µM, and 50 mM, respectively). All assays were performed using the Agilent Seahorse XFe24 analyzer and the Wave 2.6.3. software.

### Statistical analyses

Data were analyzed with paired or unpaired t-tests and one- or two-way ANOVA, using Prism 8 Software. Data are expressed as mean ± standard deviation (SD) or standard error (SEM) and are representative of at least three independent experiments. An error with a probability of *p* < 0.05 was considered significant. Detailed statistical analyses for each experiment are reported in Figure legends.

Additional methods are provided in Supplemental Information.

## Results

### Transcriptomic analyses of RS and CLL cells reveal metabolic genes as differentially expressed between the two disease phases

We first compared the transcriptomic profiles of four primary RS samples, their corresponding RS-PDX-derived cells (RS1316, RS9737, RS1050, IP867/17) [15, 16], and a cohort of primary CLL patients, whose RNA-Seq data were publicly available [18].

**Fig. 1:**
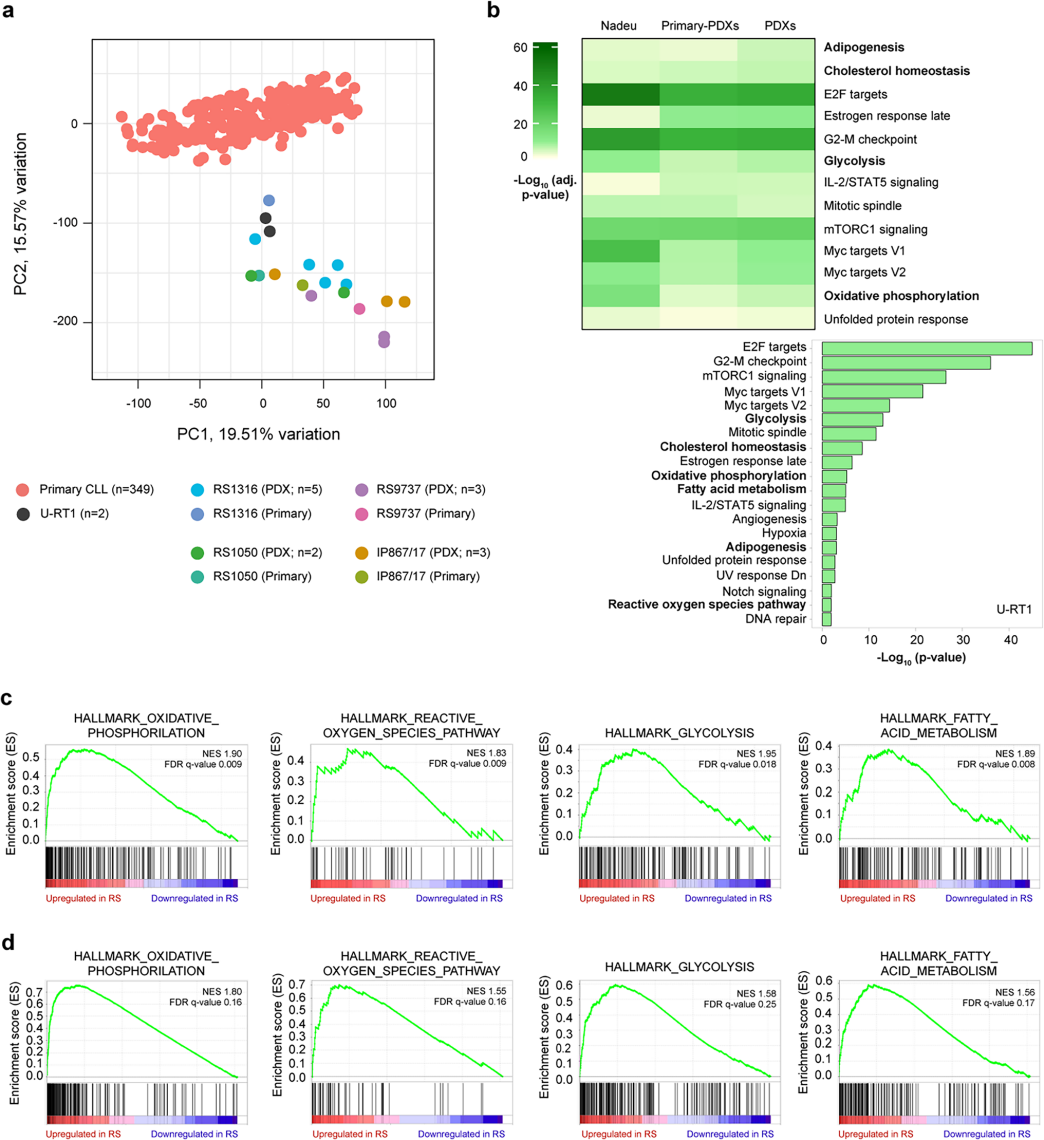
Transcriptomic analyses of RS and CLL cells reveal metabolic genes as differentially expressed between the two disease phases. **a** PCA shows the distribution of U-RT1 (n = 2), RS-PDXs (n = 13), their corresponding primary samples (n = 4), and primary CLL samples (n = 349). U-RT1 clusters together with RS models are clearly separated from CLL samples. PC1 and PC2 account for 19.51% and 15.57% of the variance, respectively. CLL data were obtained from publicly available datasets (GSE92626, GSE66117, GSE176141, GSE119103, EGAD00001004046, and EGAD00001000258). **b** Heatmap showing the first most significant gene ontology (GO) terms based on MSigDB Hallmark 2020 database found to be enriched both in a cohort of primary RS samples^18^ and in our cohort of PDX and their corresponding primary samples. –log_10_ of the adjusted p-value of each GO term is plotted (upper panel). Histogram showing the first 20 most enriched GO terms of up-regulated genes in the U-RT1 cell line (3 biological replicates) versus primary CLL samples (n = 10). All 13 samples were obtained from publicly available microarray data (U-RT1: GSE171481, primary CLL: GSE75122; lower panel). **c** Gene set enrichment analysis (GSEA) of PDX-corresponding primary samples versus primary CLL ones, showing the enrichment of oxidative phosphorylation, reactive oxygen species, glycolysis, and fatty acid metabolism. Both normalized enrichment scores (NES) and FDR q-values are shown in each plot. **d** GSEA of PDXs vs primary CLL samples. The same metabolic pathways as panel c are shown

Principal component analysis (PCA) showed a clear separation between CLL and RS samples, underlining that disease transformation is accompanied by profound changes in the transcriptomic landscape (Fig. 1a). Consistent data were obtained from the profile of U-RT1, the only available RS cell line (Fig. 1a), which cluster together with RS samples [17]. We then compared our in-house generated RNA-Seq analyses, performed both on primary RS samples and their corresponding PDXs, with a recently published cohort of six CLL patients who transformed to RS [19]. OXPHOS and glycolysis appeared to be enriched in RS samples (Fig. 1b, upper panel). Microarray analyses performed on U-RT1 confirmed the upregulation of these metabolic pathways in RS compared to CLL, underscoring also the transcriptomic similarity among the different RS models (Fig. 1b, lower panel).

In line with these results, gene set enrichment analysis (GSEA) comparing either primary RS (Fig. 1c and Table S1) or PDXs (Fig. 1d and Table S2) versus primary CLLs clearly showed enrichment in several metabolic pathways, confirmed also by an over-representation analysis (ORA; Supplemental Fig. S1a, b). The separation between the two disease phases from the metabolic perspective was further highlighted by PCA performed specifically on genes belonging to OXPHOS, reactive oxygen species, glycolysis, and glutamine/glutamate metabolism (Supplemental Fig. S1c–g).

These data indicate that RS transformation is paralleled by metabolic rewiring to sustain the higher proliferation rate of these cells, underscoring the need for a deeper characterization of their metabolic profile. Moreover, they suggest that PDXs and U-RT1 cells can recapitulate the main features of primary samples, as highlighted also by a conserved transcriptomic profile of metabolic genes (Fig. S1h), and can therefore be used for a more in-depth characterization.

### RS-PDX models are metabolically different from CLL patients

**Fig. 2:**
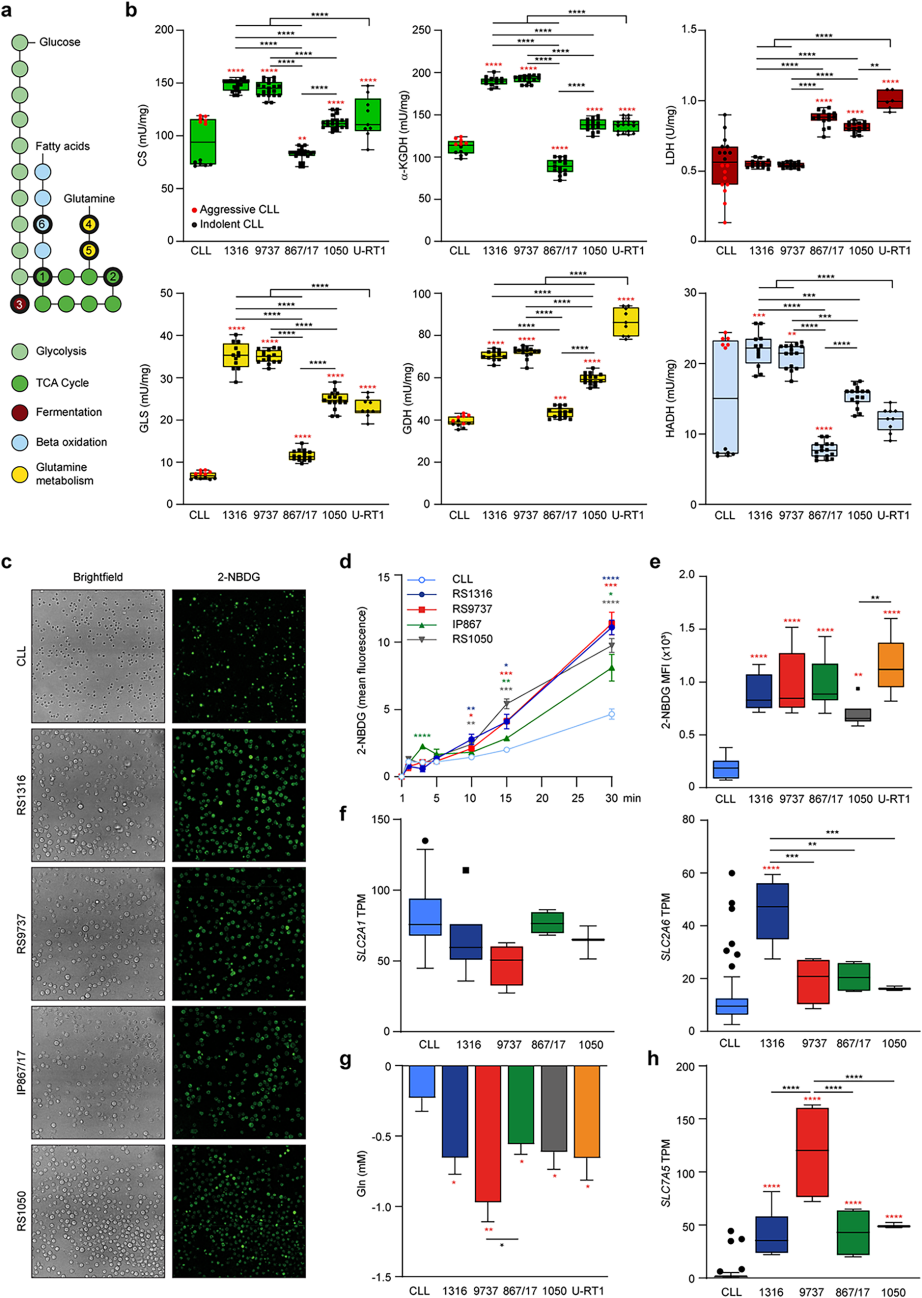
RS cells show different metabolic activities compared to CLL patients. **a** Schematic metabolic map depicting the main metabolic pathways analyzed. Bold edges highlight the investigated enzymes. Citrate synthase (1; CS); oxoglutarate (Alpha-Ketoglutarate) dehydrogenase (2; α-KGDH); lactate dehydrogenase (3; LDH); glutamine synthetase (4; GLS); glutamate dehydrogenase (5; GDH); hydroxyacyl-CoA dehydrogenase (6; HADH). **b** Cumulative data (box plot) showing the activity of selected metabolic enzymes measured in primary CLL or RS-PDX-derived cells. For RS, each RS-PDX model (RS1316; RS9737; IP867/17; RS1050) and U-RT1 cell line are independently plotted to highlight potential differences among them (data from 3 independent experiments are shown; black squares). CLL samples are plotted as indolent (black dots) or aggressive (red dots) patients (3 patients each in duplicate, except for LDH which was performed in triplicate). **c** Representative images of glucose internalization measured by confocal microscopy in live CLL or RS cells using the fluorescent compound 2-NBDG (30 min incubation; magnification 40X). **d** Quantification of confocal 2-NBDG mean fluorescence intensity (MFI) from 3 independent experiments. For each sample, the MFI was measured in each single cell in 4 independent fields. Statistically significant differences between RS and CLL samples are reported. **e** 2-NBDG MFI measured by cytofluorimetric analyses after 30 min of staining. **f** Box plot showing the transcript per million (TPM) of selected glucose transporters (GLUT1/*SLC2A1* and GLUT6/*SLC2A6*) in CLL patients and RS-PDX models. **g** Bar plot showing the glutamine uptake of CLL and RS cells. **h** Box plot showing the TPM of selected glutamine transporter (LAT1/*SLC7A5*) in CLL patients and RS-PDX models. For panels **e**–**h**, data are reported as mean ± SEM. Statistical analysis was performed using 1-way analysis of variance (ANOVA); *P 0.05, **P 0.01, ***P 0.001, ****P 0.0001; red asterisks summarize statistical significance between RS and CLL, while black ones show statistical significance among RS-PDX models, including the U-RT1

Given the key role of metabolism in cancer cell biology and its changes during RS transformation, we evaluated the activity of critical enzymes belonging to glucose, fatty acid, and glutamine metabolism (Fig. 2a). First, we analyzed pathways involved in glucose catabolism, considering two processes downstream of glycolysis: the TCA cycle, indicative of cellular respiration, and fermentation, indicative of the Warburg effect. The activities of citrate synthase (CS) and alpha-Ketoglutarate dehydrogenase (α-KGDH) were significantly higher in RS1316, RS9737, RS1050, and U-RT1 compared to CLL, while IP867/17 exhibited enzyme activity comparable to primary CLL cells (Fig. 2b). A similar pattern was observed for glutaminolysis, where the enzymatic activities of GLS and GDH were higher in all PDXs and the U-RT1, with some heterogeneity, except for IP867/17, which was more like CLL, suggesting that glucose is not the sole energy source for RS cells (Fig. 2b). Conversely, lactate dehydrogenase (LDH) activity was stronger in U-RT1 and IP867/17, followed by RS1050, compared to RS1316, RS9737, and CLL, suggesting that the former models may also utilize lactate fermentation to regenerate NAD^+^ (Fig. 2b). The higher LDH activity observed in IP867/17 and RS1050 was confirmed by histochemical detection of enzymatic activity in tumor masses derived from RS-PDXs (Fig. S2a). These data indicate that the enzymatic activities measured in RS cell suspensions closely reflect the active metabolism in tissues. Overall, these results indicate that three out of four models and the U-RT1 preferentially use the TCA cycle and glutamine metabolism for energy production. Additionally, when dividing CLL patients into aggressive (advanced CLL stage, unmutated *IGVH* gene, mutated *TP53* and/or unfavorable cytogenetics, CD38 positivity; Table S3) or indolent (at the time of analysis), U-RT1, RS1316, RS9737, and RS1050 are more closed to the aggressive subset.

Gene and protein expression of the main enzymes analyzed above were then examined in both RS and CLL (Table S4). RS invariably displayed higher mRNA levels than CLL cells for all tested enzymes, in agreement with transcriptomic data (Fig. S2b), except for *GLS* in U-RT1. At the protein level, a more homogenous expression for all the enzymes was observed between the two disease phases, except for LDHA (Fig. S2c). When CLL samples were divided into indolent and aggressive patients, the aggressive ones exhibited enhanced enzyme expression compared to the indolent counterparts (Fig. S2d).

Finally, we focused on the uptake of glucose and glutamine by RS cells. All the RS-PDXs exhibited enhanced glucose uptake compared to CLL, as shown by confocal microscopy (Fig. 2c, d) and flow cytometry (Figs. 2e and S3a) analyses. Specifically, kinetic experiments highlighted a significant increase in RS cells starting at 10 min of incubation with 2-NBDG and peaking at 30 min, demonstrating that RS cells are more avid for glucose compared to leukemic cells. No significant differences in GLUT1/*SLC2A1* transporter gene expression were detected (Fig. 2f), but the higher glucose uptake observed in RS cells correlated with a significantly higher expression of *SLC2A6,* encoding the hexose transporter (Fig. 2f). Additionally, increased expression of other glucose transporters was noted for RS1050 (*SLC2A5*) and IP867/17 (*SLC2A4;* Supplemental Fig. S3b). Glutamine uptake, measured as a decrease of the substrate in the culture media, was also significantly enhanced in RS cells compared to CLL, with RS9737 showing the highest glutamine reduction in the medium after 24 h (Fig. 2g). The glutamine uptake profile observed in RS cells correlated with a significantly higher expression of *SLC7A5, SLC1A5,* and *SLC38A5*, all neutral amino acid transporters, in all RS-PDX models (Figs. 2h and S3c). Expression at the mRNA levels of glucose and glutamine transporters was confirmed also at the protein level (Fig. S3d, e).

### Energy status and biosynthetic pathways activation in RS cells

**Fig. 3:**
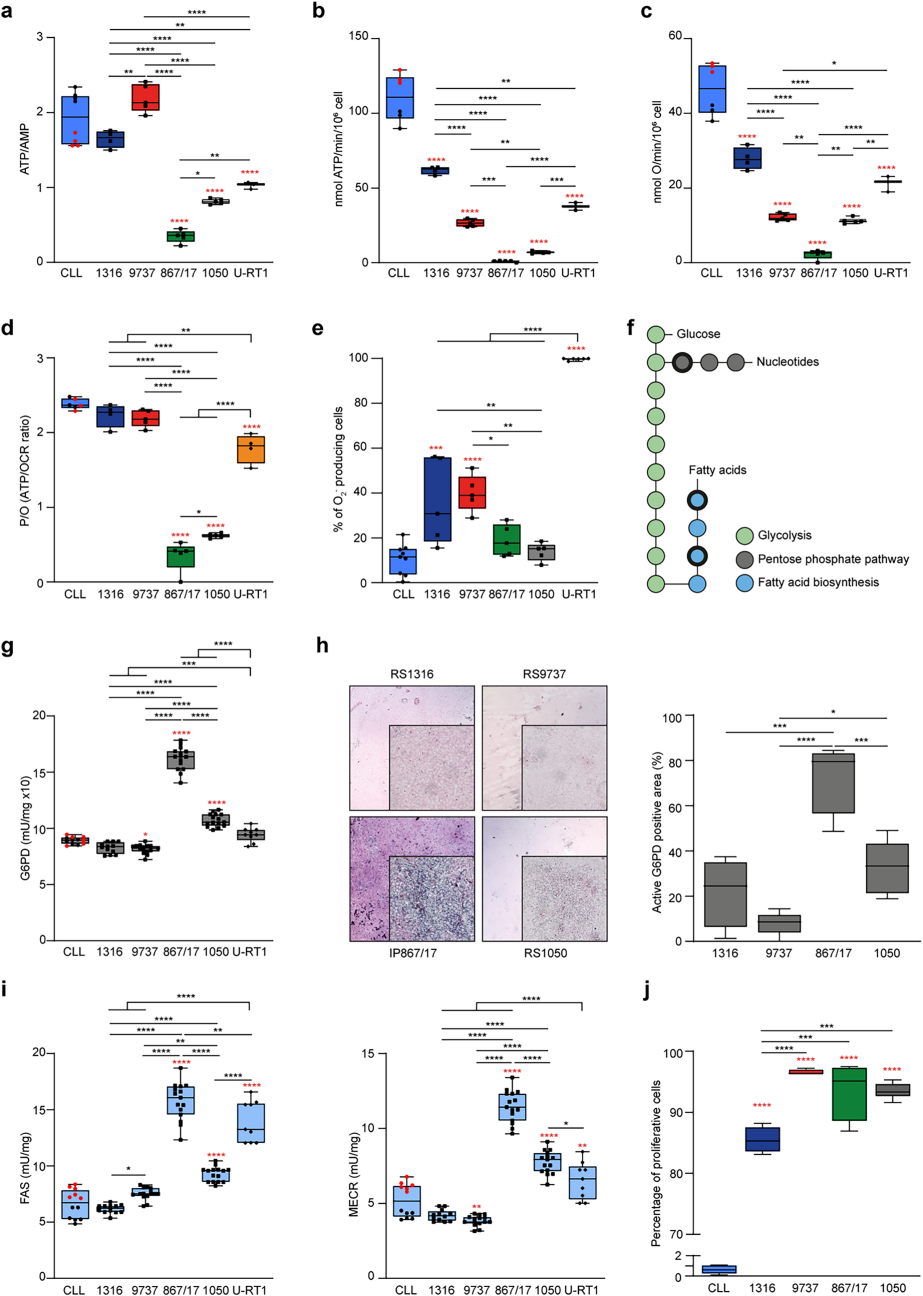
RS cells metabolic activity leads to ATP production and biosynthetic pathways activation. **a**–**c** Energetic status and mitochondrial activity of CLL and RS-PDX-derived cells and U-RT1 represented as ATP/AMP ratio (**a**), aerobic ATP synthesis (molecules per million cells (**b**) and oxygen consumption rate (OCR; **c** Data reported in **b** and **c** were obtained using 10 mM pyruvate and 5 mM malate as respiratory substrates. **d** P/O value, calculated as the ratio between synthesized ATP and consumed oxygen, an OXPHOS efficiency marker. **e** Flow cytometry analysis of the electron transport chain activity in CLL and RS cells, measuring O_2_^−^ production with MitoSOX kit. Data are shown as the percentage of positive O_2_^−^ producing cells. **f** Schematic metabolic map showing the main anabolic pathways starting from glucose. Bold edges highlight the investigated enzymes. **g** Box plots showing the activity of the enzyme G6PD, involved in the pentose phosphate pathway. **h** Histochemical detection of the enzymatic activity of the glucose-6-phosphate dehydrogenase (G6PD; representative images) in tumor masses sections of RS-PDX models (magnification X4; inset X40). Box plot represents the percentage of G6PD activity positive area in 5 independent experiments. **i** Box plots showing the enzymatic activity of the fatty acid synthase (FAS) and mitochondrial trans-2-enoyl-CoA reductase (MECR) both involved in fatty acid biosynthesis. **j** Proliferation rate of CLL and RS cells measured by flow cytometry based on Ki-67 staining. Box plots report the percentage of Ki-67^+^ cells. In panels showing the enzymatic activities of selective enzymes, CLL samples are plotted as indolent (black dots) or aggressive (red dots) patients. Statistical analysis was performed using 1-way analysis of variance (ANOVA); *P 0.05, **P 0.01, ***P 0.001, ****P 0.0001; red asterisks summarize statistical significance between RS and CLL, while black ones show statistical significance among RS-PDX models, including the U-RT1

We then focused on the energy status of RS cells and the activation of biosynthetic pathways. RS1316, RS9737, RS1050, and U-RT1 were characterized by a favorable energy status, as indicated by the ATP/AMP ratio (Fig. 3a), which correlated with higher mitochondrial ATP production (Figs. 3b and S4a) and oxygen consumption rate (OCR; Figs. 3c and S4b), albeit with some heterogeneity among them. In contrast, IP867/17 exhibited the lowest ATP/AMP ratio (Fig. 3a), correlating with minimal mitochondrial ATP production (Figs. 3b and S4a) and oxygen consumption (Figs. 3c and S4b). Furthermore, the P/O value, calculated as the ratio between synthesized ATP and consumed oxygen, indicated that RS1316, RS9737, and U-RT1 were characterized by coupled respiratory metabolism, where all consumed oxygen is used for energy production. In contrast, RS1050 and IP867/17 exhibited uncoupled respiratory metabolism, where part of the consumed oxygen is not used for energy production but contributes to ROS formation (Fig. 3d). These results were supported by the high production of the ion superoxide, directly linked to the electron transport chain function (Fig. 3e) [20]. Differences in OXPHOS activity among the models did not correlate with the number of mitochondria within the cells (Supplemental Fig. S4c), suggesting that mitochondrial defects may be present in RS1050 and IP867/17 or genetic/epigenetic factors may control their activity. Notably, CLL cells are characterized by high levels of ATP, consistent with a metabolism based on fatty acids, which results in higher chemical energy production (Fig. 3a–c).

Despite the generation of oxidative stress, which is detrimental to the cell, oxidative damage accumulation also depends on the endogenous antioxidant response. Data obtained by measuring catalase (CAT) and glutathione reductase (GRX) activities showed that RS cells had a similar range of antioxidant system activation, except for IP867/17, which exhibited higher levels of CAT and GRX activities (Fig. S4d), consistent with their gene expression (Fig. S4e). However, evaluating malondialdehyde (MDA) levels as a marker of lipid peroxidation revealed that all RS samples displayed a higher MDA concentration than CLL, with RS1050 and U-RT1 showing the highest values (Fig. S4f). These data suggest that despite the activation of antioxidant defense, RS samples accumulate oxidative damage.

Next, we evaluated anabolic processes, including the pentose phosphate pathway (PPP) and fatty acid biosynthesis (Fig. 3f). Glucose-6-Phosphate Dehydrogenase (G6PD) activity was comparable between CLL, U-RT1, and RS-PDXs, except for IP867/17, which exhibited higher activity (Fig. 3g), confirmed by histological metabolic staining (Fig. 3h). A similar trend was observed for fatty acid synthase (FASN) and Mitochondrial Trans-2-Enoyl-CoA Reductase (MECR) activities, with IP867/17 showing a higher metabolic rate of fatty acid synthesis compared to the other RS-PDXs, U-RT1 and CLL (Fig. 3i). The activity of these enzymes was independent of expression levels, which generally highlighted higher expression in RS cells compared to CLL (Fig. S5a, b).

The higher metabolic activity measured in RS cells, necessary to sustain the higher proliferation rate that characterizes these lymphoma cells (Fig. 3j), may be the result of disease progression from one phase to the other and should reflect changes at the genomic and epigenetic levels [19].

### Metabolomic and transcriptomic data confirm the activation of energetic and biosynthetic pathways in RS cells

**Fig. 4:**
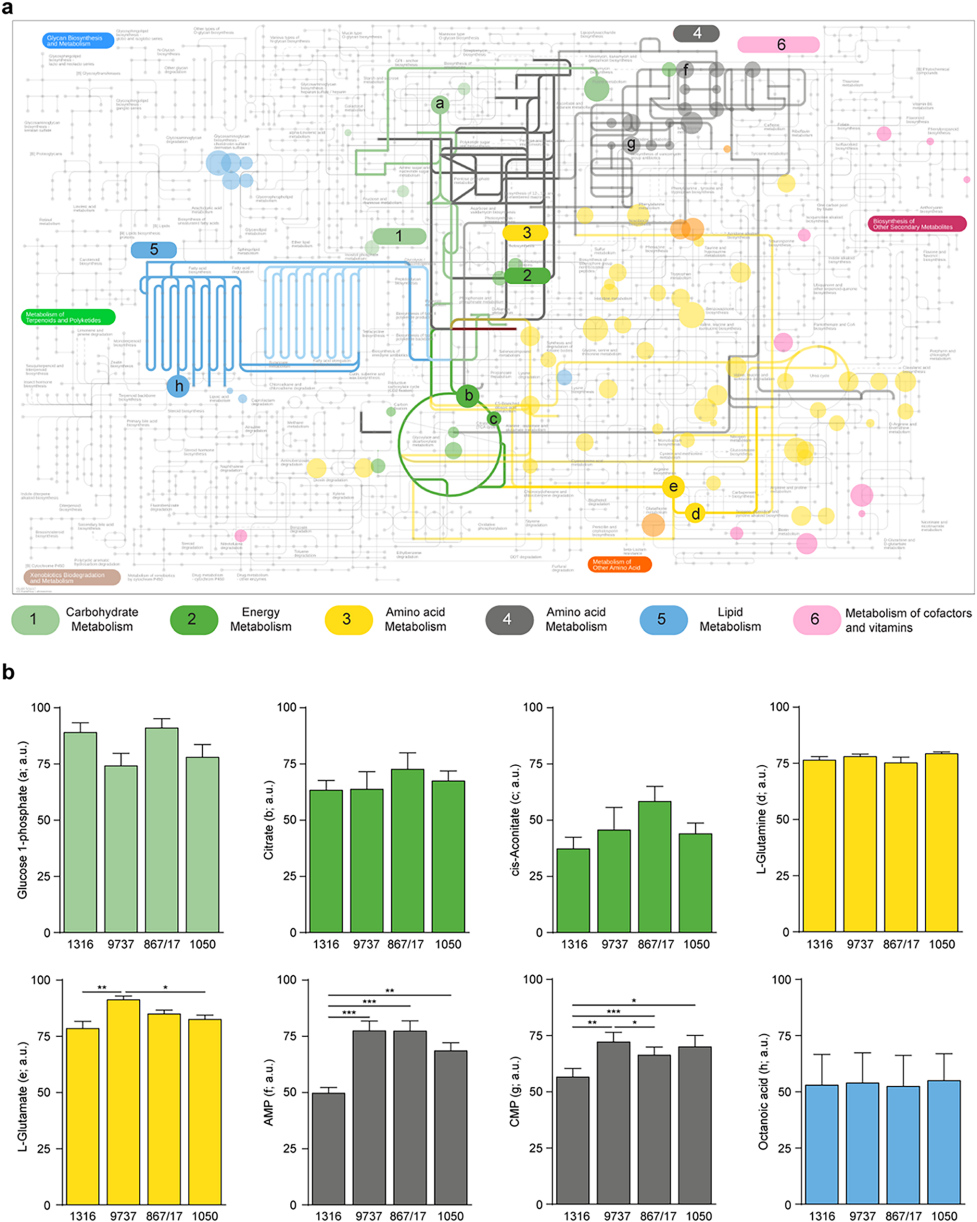
Metabolomic analyses of RS cells. **a** Schematic map obtained using the iPath3.0 software reporting the cellular metabolic network and the metabolites (colored dots) measured using an untargeted HRMS metabolomic approach. In bold, with a color code, are shown the main metabolic pathways active in RS cells (green: carbohydrate and energy metabolism; grey: nucleotides metabolism; yellow/orange: amino acids metabolism; light blue: lipid metabolism). **b** Bar plots showing the quantification of the indicated metabolites (among the most representative ones measured by the metabolomic assay) in 3 independent experiments. The area under the chromatogram peak, proportional to the metabolite quantity in each sample, was calculated and reported as mean ± SEM expressed in arbitrary units (a.u.). The proportionality between peak area and metabolite quantity allows for relative comparison of metabolite levels across the different experiments. Statistical analysis was performed using 1-way analysis of variance (ANOVA); *P 0.05, **P 0.01, ***P 0.001

To detect endogenous metabolites and highlight th, active pathways, an untargeted high-resolution mass spectrometry metabolomic analysis was performed. Results showed a relatively small number of detectable metabolites within RS cells with minimal differences among PDXs, suggesting that despite some differences in the metabolic activity of specific enzymes, these cells share common features. Based on the metabolomic map derived from these data, several conclusions can be drawn (Fig. 4a). First, energy metabolism is active, as evidenced by the presence of metabolites sustaining the glycolytic pathway, which in turn feeds the TCA cycle, indicated by the presence of citrate and cis-aconitate (Fig. 4b). The TCA cycle is also sustained by glutamine and glutamate, two other relevant metabolites measured in RS cells (Fig. 4a, b). Additionally, RS cells are characterized by an active antioxidant scavenger system, represented by the pentose phosphate pathway (PPP), which contributes to the restoration of NADPH. Furthermore, RS cells showed activation of anabolic pathways, including the PPP, nucleotides and amino acid metabolisms, and lipid biosynthesis, resulting in the production of building blocks necessary for nucleic acids, proteins, and lipid synthesis (Fig. 4a, b).

**Fig. 5:**
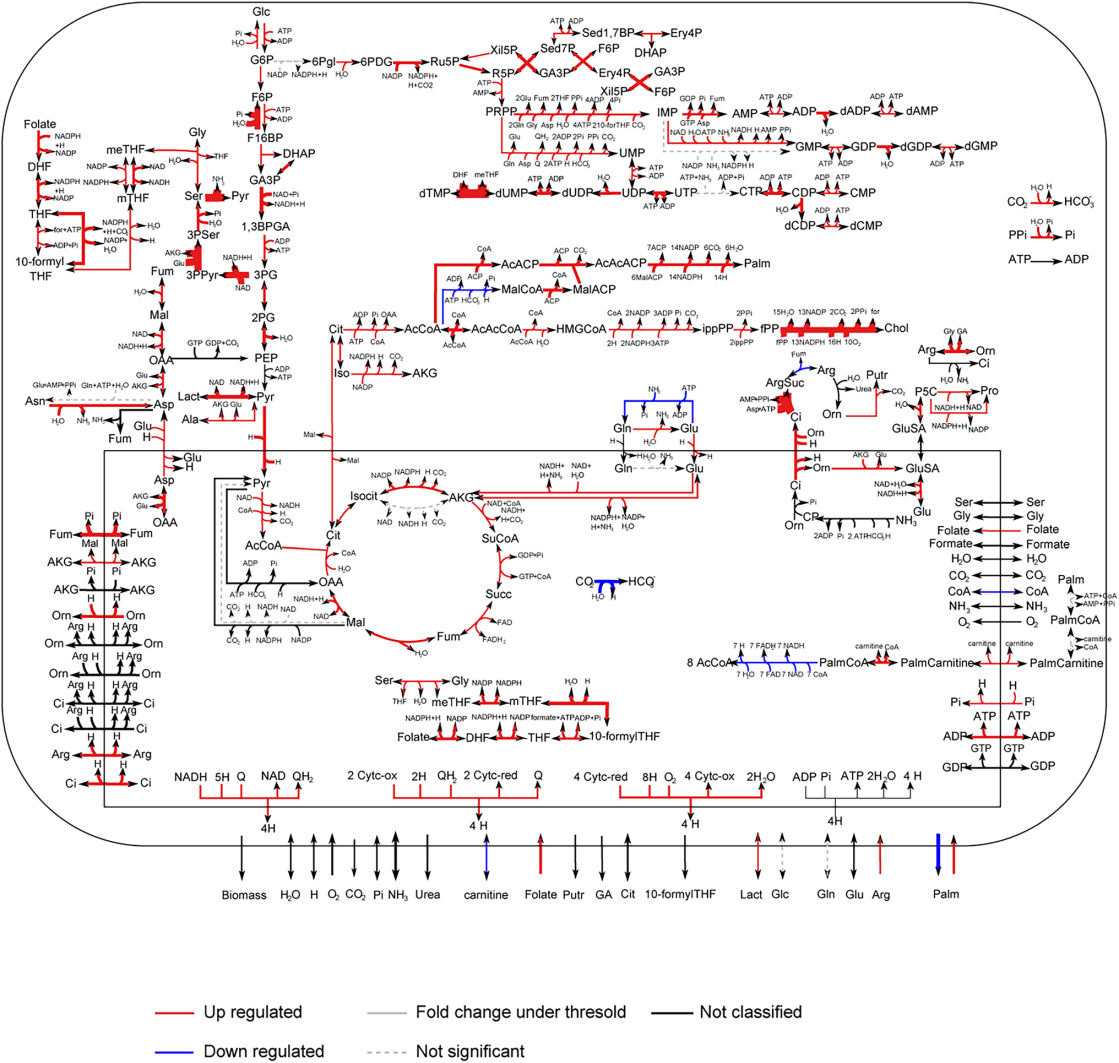
Metabolic map of RS cells based on transcriptomic data. Metabolic map, obtained using the Marea4Galaxy software, comparing RS-PDX to CLL cells. Red arrows indicate reactions that are up-regulated, whereas blue arrows indicate reactions that are down-regulated. Black arrows refer to reactions with no info available. Dashed grey arrows refer to non-significant dysregulations according to the Kolmogorov–Smirnov test with a *p*-value of 0.01. Solid grey arrows refer to reactions with a variation lower than 20%

Taken together, these data suggest that RS transformation is paralleled by the activation of several pathways necessary to sustain the energy demands and high proliferation rate. Further support for these conclusions was obtained by re-analyzing transcriptomic data comparing RS to CLL, focusing selectively on metabolic-related transcripts, and generating a “transcriptomic metabolic map” (Fig. 5). This analysis confirmed the activation of energetic pathways that sustain the production of ATP (glycolysis and glutaminolysis), as well as anabolic processes that lead to the synthesis of nucleotides and cellular membrane components (Fig. 5). At the same time, several terms related to negative regulation of metabolic pathways (e.g., negative regulation of macromolecules biosynthetic process, negative regulation of cellular biosynthetic process, negative regulation of fatty acid metabolic process, cellular response to glucose starvation) were significantly down-regulated (data not shown).

### RS cells are strongly dependent on glucose and glutamine

**Fig. 6:**
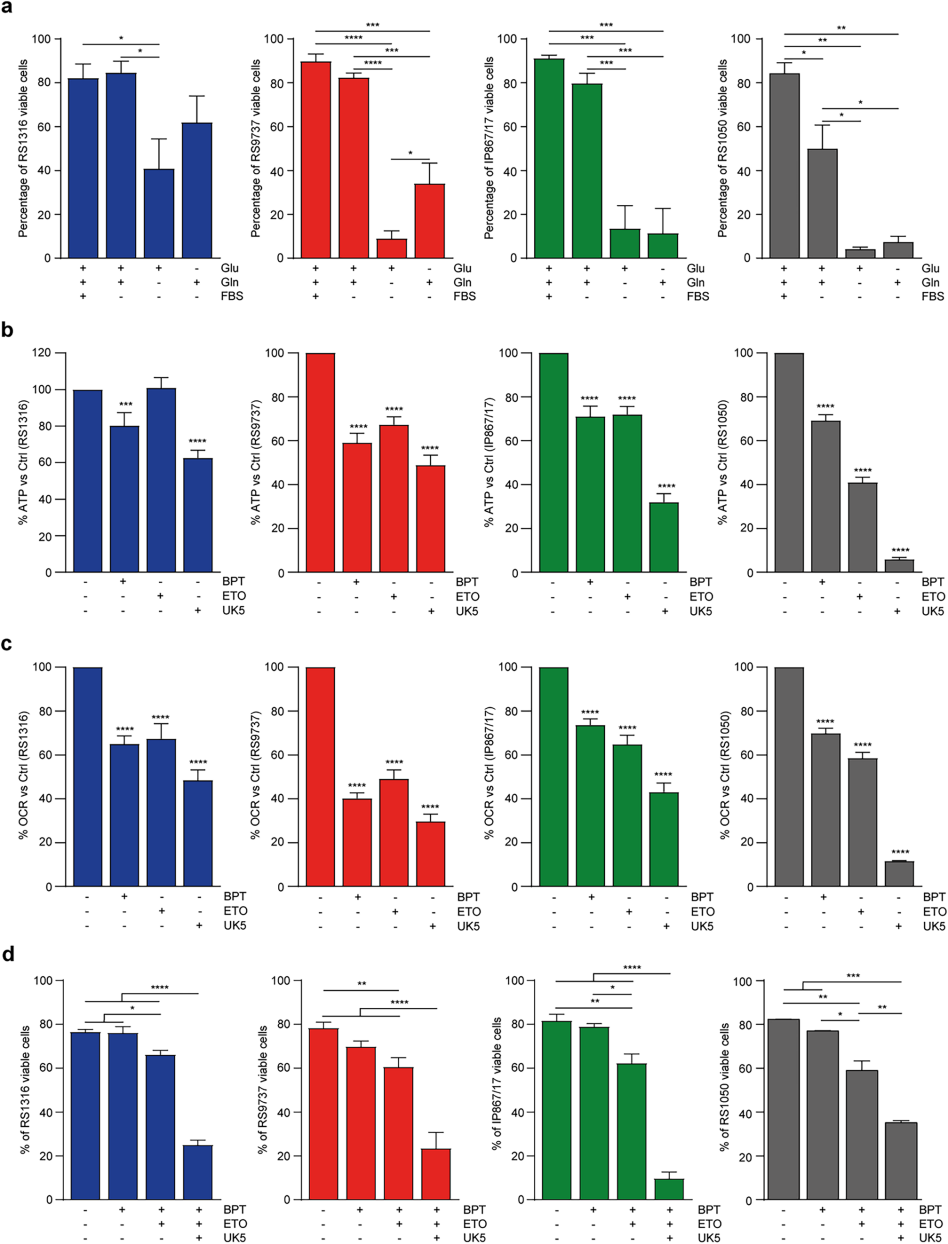
RS-PDX models metabolic dependencies. **a** Apoptotic assay measuring the substrate dependencies (glucose: Glu; glutamine: Gln; fatty acid: fetal bovine serum FBS which is enriched in fatty acids) of RS cells. Bar plots report the percentage of viable cells after 48 h of culture in complete or substrate-deprived media. **b**, **c** ATP production (**b**) and O_2_ consumption (**c**) by RS cells in the absence or presence of selective metabolic inhibitors (BPTES/BPT: glutaminase inhibitor; Etomoxir/ETO: carnitine palmitoyl-transferase 1 inhibitor; UK5099/UK5: mitochondrial pyruvate carrier inhibitor). Data are shown as the percentage of residual activity compared to the untreated condition. **d** Apoptotic assay showing the percentage of viable RS cells after 48 h of treatment with BPT, ETO, and UK5. Data are reported as mean ± SEM. Statistical analysis was performed using 1-way or 2-way analysis of variance (ANOVA); *P 0.05, **P 0.01, ***P 0.001, ****P 0.0001

We then focused on the metabolic dependencies of RS cells to determine the substrates these cells rely on. Firstly, RS cells were cultured in selective culture media supplemented with glucose, glutamine, and/or FBS (as a provider of fatty acids). The absence of glucose and glutamine significantly impacted cell viability after 48 h of culture in all models tested, underlining their strong dependency on these two substrates (Fig. 6a). These findings were confirmed by measuring ATP production and OCR in RS cells in the presence of selective metabolic inhibitors: BPTES, Etomoxir, and UK5099. Exposure to these compounds resulted in a pronounced reduction in ATP production (Figs. 6b and S6a–S7a) and OCR (Figs. 6c and S6b–S7b), with the strongest effect obtained with the mitochondrial pyruvate carrier inhibitor for PDXs and with the glutaminase inhibitor for U-RT1. These results indicate that during transformation, RS cells are less dependent on fatty acids compared to CLL cells (Fig. S6c) as they shift towards an increased use of glutamine and glucose. Consistently, exposure to Etomoxir did not significantly affect cell viability. Interestingly, even BPTES did not result in any decrease in cell viability, suggesting that RS cells can survive primarily through glucose metabolism. However, when both glutamine and glucose pathways were inhibited, a significant drop in cell viability was measured (Fig. 6d). Unlike PDXs, the U-RT1 cell line appeared to be more resistant to substrate deprivation, as indicated by reduced differences in cell viability after glucose or glutamine removal from the culture media (Fig. S7d).

**Fig. 7:**
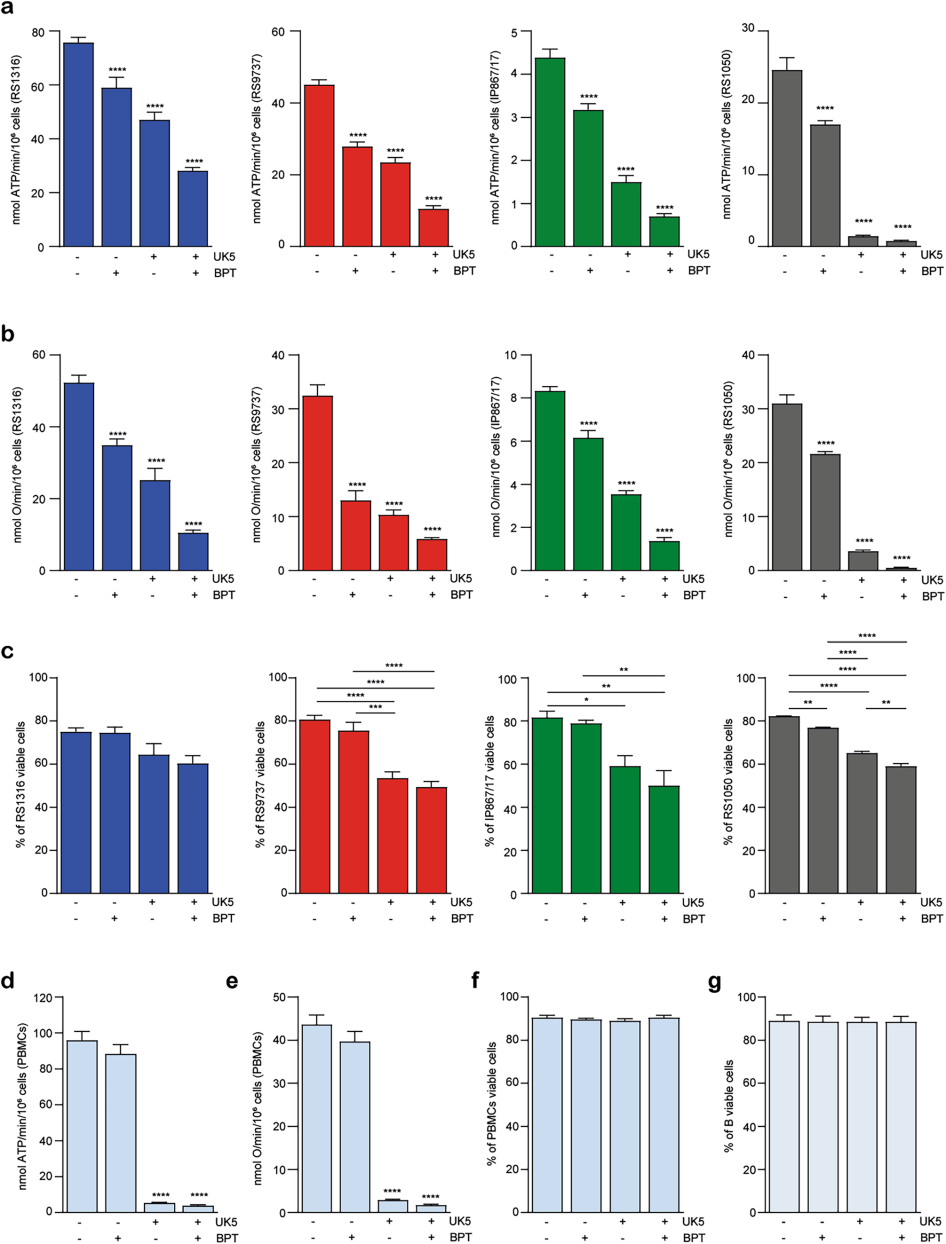
Dual Inhibition of Glucose and Glutamine Metabolism in RS cells and PBMCs.** a**, **b** ATP production (**a**) and O_2_ consumption (**b**) by RS cells in the absence or presence of glutamine (BPTES/BPT: glutaminase inhibitor) and glucose (UK5099/UK5: mitochondrial pyruvate carrier inhibitor) selective inhibitors, alone or in combination, plotted as nmol per minute (min) per million cells. **c** Apoptotic assay showing the percentage of viable RS-PDXs after 48 h of treatment with BPTES (BPT, 4 µM) and UK5099 (UK5, 4 µM), alone or in combination. **d**, **e** ATP production (**d**) and O_2_ consumption (**e**) by peripheral blood mononuclear cells (PBMCs) from healthy donor cells in the absence or presence of glutamine (BPTES) and glucose (UK5099) selective inhibitors, alone or in combination, plotted as nmol per minute (min) per million cells. **f**, **g** Apoptotic assay showing the percentage of viable PBMCs (**f**) and B cells (**g**) after 48 h of treatment with BPTES (BPT, 4 µM) and UK5099 (UK5, 4 µM), alone or in combination. Data are reported as mean ± SEM. Statistical analysis was performed using t-test or 1-way analysis of variance (ANOVA); *P 0.05, **P 0.01, ***P 0.001, ****P 0.0001

Overall, these results confirmed the strong dependency of RS cells on glucose and glutamine metabolism, presenting potential translational targeting options. In line with this view, RS cells showed a dramatic decrease in ATP production and OCR when both these pathways were targeted (Fig. 7a, b), with a parallel reduction in cell viability (Fig. 7c). On the contrary, peripheral blood mononuclear cells from healthy donors only showed a significant impact on ATP production and OCR when exposed to UK5099, suggesting that pyruvate is the principal aerobic substrate for these cells (Fig. 7d, e) [21, 22]. Interestingly, the overall impact on cell viability was not significant when healthy PBMCs were exposed to both metabolic inhibitors, favoring the hypothesis that both B and T cells rely on glycolysis and lactate fermentation for their energy demand (Fig. 7f, g) [21–23].

### Targeting PI3K and NF-kB diminishes OCR and ECAR in RS cells

**Fig. 8:**
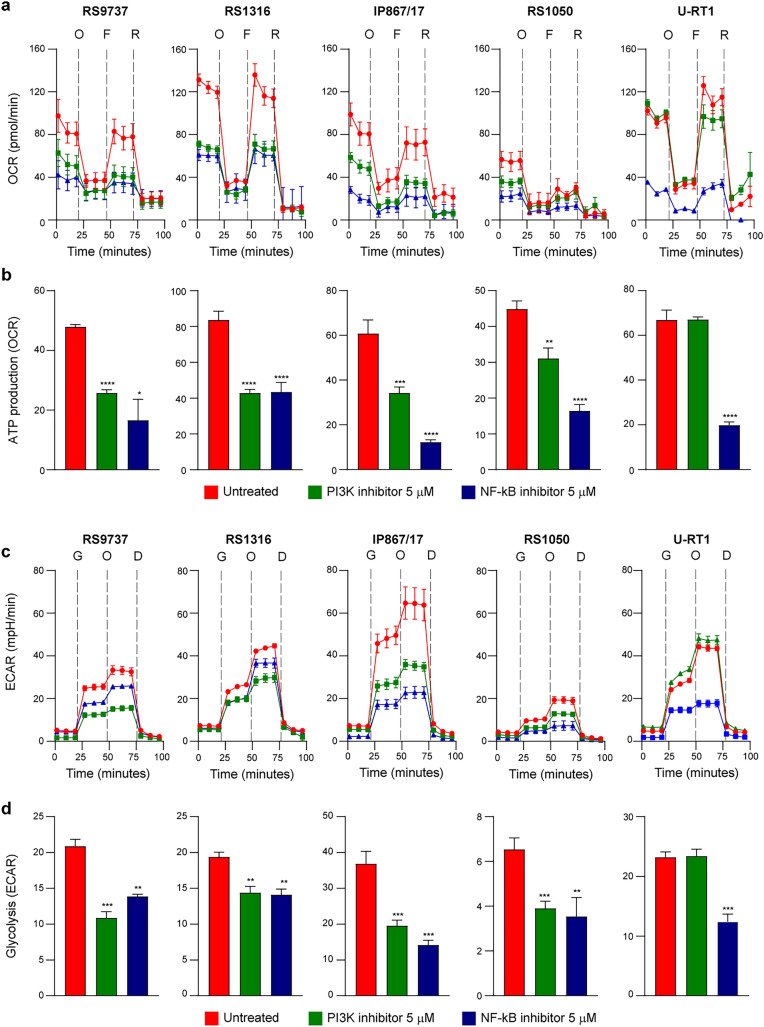
Impact of PI3K and NF-kB targeting on RS cells metabolism.** a** Cumulative profiles of OCR in the absence (red line) and presence of PI3K (Duvelisib; 5 µM; green line) or NF-kB (SC75741; 5 µM; blue line) inhibitors, after 24-h treatment. Data were obtained with Seahorse XF-24 Extracellular Flux Analyzer. Dotted lines indicated the injection of mitochondrial inhibitors oligomycin (O; 1.5 μM), FCCP (F; 0.25 μM) and rotenone/antimycin (R; 0.5 μM). **b** Histograms showing ATP production by the mitochondria measured as the difference in OCR before and after oligomycin injection. **c** Cumulative profiles of ECAR, obtained with Seahorse XF-24 Extracellular Flux Analyzer, in absence (red line) and in the presence of PI3K (green line) or NF-kB (blue line) inhibitors, after 24-h treatment. Dotted lines indicated the injection of glucose (G; 10 mM), oligomycin (O; 1 μM) and 2-DG (D; 50 mM). **d** Histograms showing the glycolysis level, measured as the difference in the ECAR before and after glucose injection. Data are reported as mean ± SEM. Statistical analysis was performed using t-test; *P 0.05, **P 0.01, ***P 0.001, ****P 0.0001

Given that RS cells are strongly dependent on OXPHOS and glycolysis, we explored the impact of targeting PI3K and NF-kB, two master metabolic regulators within the cells [24, 25] and therefore worthy to be exploited as targets [26–28]. RS cells were exposed for 24 h to either duvelisib, a dual PI3Kγ/δ inhibitor, or to SC75741, a NF-kB inhibitor targeting the p65 subunit, prior to real-time analysis of their metabolic profile using the Seahorse Analyzer. This approach allowed us to monitor OCR and ATP production (Figs. 8a, b and S8a) and ECAR (Figs. 8c, d and S8b) as measures of mitochondrial respiration and glycolytic flux, respectively. Treatment of RS cells with both inhibitors significantly impacted mitochondrial activity, resulting in an overall reduction in ATP production (Fig. 8b) and basal respiration (Fig. S8a). Similarly, treated RS cells exhibited a marked decrease in their capacity to convert glucose into pyruvate via glycolysis (Fig. 8d) and an increase in glycolysis when OXPHOS is shutdown (Fig. S8b). In contrast to RS-PDXs cells, the U-RT1 cell line showed diminished mitochondrial respiration and glycolytic flux only when exposed to the NF-kB inhibitor, with no effect in the presence of the PI3K inhibitor. One possible explanation for this result could be a copy number loss in chromosome 1p that includes the *PIK3CD* gene [17].

The impact of these inhibitors on the metabolic features of RS cells appeared to be highly selective and not due to non-specific toxicity, as indicated by the high percentage of viable cells after drug treatments (Fig. S8c, d) and previous data [29].

Overall, these data suggest that metabolic properties and dependencies of RS cells represent an Achille’s heel for the disease and can be exploited as targets.

## Discussion

Significant advancements have been made in understanding the pathogenesis of RS transformation, with some still unexplored features. This study aimed to characterize the metabolic adaptations essential to sustain disease progression and, by using RS-PDX models and a cell line, several key insights have been revealed.

First, transcriptomic analysis comparing primary RS cells, RS-PDXs, and primary CLL samples clearly distinguishes these two disease phases. Several “metabolism-related terms” were among the most differentially expressed genes between RS and CLL across various RS sample cohorts, underscoring that metabolic rewiring is a hallmark of the transformation, in line with findings also from other research groups [19, 30]. Among these terms, OXPHOS and glycolysis were prominently upregulated, indicating a higher metabolic demand to support the increased proliferation rate typical of RS cells. In addition, since OXPHOS activity is linked to oxidative stress production, its increment is associated with a higher ROS content, which favors cellular proliferation and survival. It is well established that a controlled amount of ROS exerts a pro-proliferative effect, particularly in cancer cells, as well as a significant role in regulating cell death [31, 32], although adapting to sustained oxidative stress causes an increased energetic demand [33].

Second, an in-depth analysis of RS metabolism revealed slight heterogeneity in specific metabolic features, as suggested by PCA and enzymatic activity rates, but a common “metabolic signature” emerged. All RS cells rely heavily on glucose and glutamine pathways for energy production, evidenced by metabolomics analyses and functional approaches. Indeed, assays of the enzymatic activities involved in glutamine and glucose catabolism support this finding. However, it is relevant to note that in vitro assays reflect the maximal catalytic capacity of the enzyme without considering the actual substrate availability at the cellular level. Three out of four models and U-RT1 exhibited enhanced OXPHOS, consistent with transcriptomic data. The remaining model displayed a Warburg-like phenotype, relying primarily on fermentation, aligning with literature indicating that cancer cells may use fermentation for energy production even in the presence of oxygen [21]. Additionally, RS cells showed activation of anabolic pathways, leading to the production of nucleotides and lipids, necessary for their proliferation.

Thirdly, in line with the hypothesis of a “metabolic rewiring” as the disease progresses, we showed that CLL cells are characterized by aerobic metabolism and a “fit” energy state, primarily supported by fatty acids catabolism and partially by glucose, with minimal reliance on glutamine. Since the catabolism of a single molecule of 16-carbon-atom fatty acid produces 3.5 times more ATP than glucose, CLL cells can generate more energy from fatty acid consumption than RS cells. Conversely, RS cells sustain their aerobic metabolism via glutamine and exhibit enhanced glucose uptake, which supports both energy production and anabolic pathways necessary for proliferation. In other words, it can be hypothesized that the intracellular pyruvate concentration available for mitochondria energy production may be reduced due to the need to use glucose to synthesize building blocks, such as ribose-5-phosphate via PPP, leading the cells to use glutamine as an energy substrate. On the other hand, both NADPH and ribose-5-phosphate play a pivotal role in RS cell survival and proliferation as the first guarantees an efficient antioxidant response, while the second provides sufficient nucleotide pull useful for DNA replication and energy balance.

An additional point to be considered when defining the metabolic features of leukemic cells is the role of the microenvironment or the lymphoid niche, intended both as a place where tumor cells can heavily interact with other cell types finding activation signals and as an environment with a defined metabolic substrates availability. In line with this topic, it was convincingly demonstrated that CLL cells in the LN are “metabolically different” compared to those in the circulation [13, 34, 35]. It is plausible to think that a similar consideration can be true also for RS cells that are preferentially localized within secondary lymphoid niches where all the proliferative signals are met.

Another aspect that is worth discussing is the potential role of genetic lesions and their impact in controlling the metabolic rewiring of RS cells. The obtained data allowed for speculation linking the genetics of leukemic cells and metabolic adaptation. All RS-PDXs, except IP867/17, which has few cancer-related point mutations, present a complex karyotype and genetic landscape [15, 17]. This unique model also shows a different metabolic behavior. Some altered or activated genes in RS are associated with metabolism, such as *TP53*, *KRAS*, *NOTCH1,* and *MYC* [36–38]. For instance, *KRAS* mutations in RS1316 and RS9737 correlate with high glutamine consumption consistent with studies showing KRAS-mutant cells dependence on glutamine to support the Krebs cycle [37]. Similarly, *MYC* is a key regulator of glutamine metabolism [39, 40] possibly explaining the enhanced metabolism in RS1316, RS9737, RS1050, and U-RT1. Recently, Iyer and colleagues have demonstrated that *MGA* deletion, a *MYC* transcriptional repressor, leads to RS transformation and a profound change in mitochondria structure with consequent OXPHOS increase and metabolic reprogramming [30]. On the same line, *NOTCH1* appears to be implicated in driving a metabolic shift towards OXPHOS and glutamine when mutated (manuscript in preparation). On the other hand, it is known that *TP53* variants cause both transcriptional activity defects and alterations in glucose metabolism, with different consequences depending on the type of mutation [41]. Moreover, it has been recently shown that epigenetic changes occur during RS transformation, impacting the activity of different transcriptional factors that in turn regulate genes involved in OXPHOS. These features appeared to be already present in the “early dormant RS cells”, suggesting that metabolic rewiring is an intrinsic characteristic of RS cells [19]. Overall, the link between genetic/epigenetics features and metabolic adaptations is an interesting field that needs to be further explored.

Finally, the translational relevance of these findings is significant. Indeed, the data obtained showed how critical glucose and glutamine are for RS cell viability and how targeting these substrates impacts their survival. Similarly, interfering with key metabolic regulators, such as PI3K and NF-kB, significantly affected energy production via glycolysis and OXPHOS, and treatment with selective metabolic inhibitors could be a viable therapeutic strategy. In line with this view, combinations of inhibitors targeting glucose and glutamine catabolism warrant further exploration, considering that normal B cell energy metabolism is also primarily sustained by glucose, thus requiring strategies capable of delivering inhibitors exclusively to RS cells. Moreover, combination strategies targeting metabolic pathways alongside key RS players may offer promising treatment avenues.

In conclusion, our study highlights the metabolic adaptations in RS cells and their potential as therapeutic targets, providing a foundation for future research and clinical interventions.

## Supplementary Information

Below is the link to the electronic supplementary material.

Supplementary file1 (DOCX 2063 KB)

## Data Availability

For original data, please contact tiziana.vaisitti@unito.it.
