# Supplementary material for: Complementary approaches define the metabolic features that accompany Richter syndrome transformation
Source: Cell Mol Life Sci. 2025 Apr 9;82(1):152. doi: 10.1007/s00018-025-05670-4 (PMC11982009; doi:10.1007/s00018-025-05670-4)
Supplement: Supplementary file 1 — Supplementary file1 (DOCX 2063 KB) [file 18_2025_5670_MOESM1_ESM.docx]

**Supplemental information**

**RNA-Seq and transcriptomic data analysis**

After sequencing (NextSeq 550, Illumina) reads were filtered and mapped to hg19 genome using the dedicated apps on the Illumina BaseSpace Sequence Hub. Differentially expressed genes (DEG) with a |log2FC|>2 and an adjusted p-value <0.05 (method=’fdr’) were considered when comparing RS-PDX and RS-PDX-primary samples (RS primary cells which the RS-PDX derived from) to publicly available CLL data (EGAD00001004046) [1]. Up-regulated RS genes were then investigated through a gene ontology (GO) enrichment analysis by using the enrichR v3.2 R package. The MSigDB Hallmark 2020 gene set libraries were utilized for the analysis. The same analysis was run for publicly available RS samples [2], by taking the genes the authors considered to be up-regulated. GO results were then compared as follows: the first 20 most significant terms in Nadeu’s cohort having an adjusted p-value <0.05, were also searched and compared to the significant terms (adj.p-value<0.05) in the PDX and PDX’s primary cohorts. Afterwards, -log10(adj-p.values) and respective enriched terms were plotted as a heatmap by the ComplexHeatmap v2.16.0 R package, comparing the significance of the terms among the cohorts. Other R packages for the analysis comprised tibble v3.2.1, dplyr v1.1.3, and circlize v0.4.15.

Cellular pathway association was assessed by gene sets enrichment analysis (GSEA) performed by the GSEA v4.3.2 software using the following parameters: human gene set, h.all.v2023.1.Hs.symbols.gmt; nperm, 1000; scoring scheme, weighted; and chip, Human_Illumina_HumanRef_8_v3_MSigDB.v7.1.chip. The TPMs of 21,283 genes (average TPM expression > 1) of both PDX and PDX’s primary were compared to publicly available CLL data (GSE92626, GSE66117, GSE176141, GSE119103, EGAD00001004046, and EGAD00001000258). As a result, only the enriched terms with an FDR q-value <0.25 were considered significant. Over-Representation Analysis (ORA) was performed to identify enriched biological pathways in differentially expressed genes (DEGs) with a log2 fold change (log2FC) > 2 and an adjusted p-value < 0.05 in both primary RS cells - from which PDX were generated - and PDX, compared to CLL. ORA was conducted using the enrichR package in R, with genes mapped to the MSigDB_Hallmark_2020 database. Pathways with a p-value < 0.05 were considered significantly enriched. Plots were generated by using ggplot2.

Principal component analysis (PCA) was conducted by using the PCAtools v2.12.0 R package. Z-score-normalized TPMs were used as input. Among the 60,240 genes (both protein and non-protein coding genes), only those with an average TPM expression >1 (n=21,283) were included in the analysis. 349 CLL samples belonging to the publicly available datasets mentioned above (GSE92626, GSE66117, GSE176141, GSE119103, EGAD00001004046, and EGAD00001000258) were included to be compared to RS-PDX, their PDX’s primary samples and U-RT1. Furthermore, PCAs considering specific metabolic pathways were also performed. The gene lists - used as inputs for the analyses - of the pathways of glycolysis, oxidative phosphorylation, reactive oxygen species, fatty acid and glutamate-glutamine metabolism were parsed from PathCards (https://pathcards.genecards.org/). The same number of samples and metrics were also used.

Raw microarray data of a RS cell line (U-RT1, GSE171481) was harvested by using the GEOquery v2.68.0 R package. Raw CLL microarray data was also harvested from a publicly available cohort (GSE75122) to be compared to the RS cell line. Differential gene expression (DGE) analysis was carried out by the limma v3.56.2 R package, normalizing both the background (method=’normexp’) and the data (method=’quantile’). Genes having a |log2FC|>2 and an adj.p-value <0.05 (method=’BH’) were considered as DEG. Afterwards, GO enrichment analysis was performed using the enrichR package (gene set library=MSigDB Hallmark 2020) and data were plotted by the ggplot2 v3.4.3 R package as histograms.

The R version used was 4.2.2 Patched (2023-02-20 r83916 ucrt), platform x86_64-w64-mingw32.

The “transcriptomic metabolic map” was generated using the MaREA4Galaxy software, using TPM (transcript per million) as input [3].

**Cell homogenate preparation for enzymatic assays**

Cells were centrifuged at 1000 rpm for 5 min, and the growth medium was removed. The pellet was washed in PBS twice and centrifuged again. The pellet was resuspended in PBS plus protease inhibitor and sonicated twice for 10 sec, in ice and with a 30 sec break to prevent the mixture from warming, using the Microson XL Model DU-2000 (Misonix Inc). Total protein content was estimated with the Bradford method [4].

**TCA cycle enzyme assay**

For each assay 50 μg of total protein were used. Citrate synthase was assayed spectrophotometrically at 232 nm following the decomposition of acetyl-CoA. The assay mix contained: Tris-HCl (pH 8), 0.17 mM oxaloacetic acid, and 0.20 mM Acetyl-CoA [5]. α-ketoglutarate dehydrogenase was assayed spectrophotometrically at 340 nm following NAD^+^ reduction. The assay medium contained: Tris-HCl (pH 7.5), 5 mM MgCl_2_, 40 mM rotenone, 2.5 M α-ketoglutarate, 0.1 mM CoA, 0.2 mM thiamine pyrophosphate, and 1 mM NAD^+^ [5].

**Lactate dehydrogenase activity assay**

Lactate dehydrogenase (LDH) activity was assayed spectrophotometrically following NADH oxidation at 340 nm. 50 μg of total protein were resuspended in the assay mix containing: Tris-HCl (pH 7.4), 1 mM pyruvate, and 0.2 mM NADH [5].

**Glutaminase and glutamic dehydrogenase activity assay**

Glutaminase activity was assayed spectrophotometrically at 340 nm following NAD^+^ reduction. The assay mix contained: Tris-HCl (pH 8), 50 mM glutamine, 5 mM NAD^+^, and 5 IU glutamic dehydrogenase [5]. Glutamic dehydrogenase was assayed spectrophotometrically at 340 nm following NADH oxidation. The assay solution contained: Tris-HCl (pH 7.4), 20 mM α-ketoglutarate, 0.15 mM NADH, and 1 mM ADP [5]. In both cases, 0.1 mM rotenone was added to inhibit the NADH oxidation from Complex I. 50 μg of total protein were used for both assays and data were normalized on the sample protein content.

**Fatty acid metabolism evaluation**

The activity of 3-hydroxyacyl-CoA dehydrogenase, used as a marker of fatty acids beta-oxidation metabolism, was assayed spectrophotometrically at 340 nm following the NADH oxidation in the presence of acetoacetyl-CoA. The reaction mix contained: 100 mM sodium phosphate (pH 6.0), 0.2 mM NADH, and 0.1 mM acetoacetyl-CoA [6].

The activities of β-ketoacyl-ACP reductase and Enoyl-ACP-reductase have been evaluated as markers of fatty acid synthesis metabolism. β-ketoacyl-ACP reductase was assayed following the NADPH oxidation at 340 nm in the presence of acetoacetyl-CoA. The assay medium contained: 100 mM sodium phosphate (pH 7.0), 1 mM 2-mercaptoethanol, 0.2 mM NADPH, and 0.1 mM acetoacetyl-CoA [6].

Enoyl-ACP-reductase was assayed following the NADH reduction at 340 nm in crotonyl-CoA presence. The assay solution contained: 100 mM sodium phosphate (pH 7.5), 0.2 mM NADH, 1 mM dithiothreitol, and 0.8 mM crotonyl-CoA. 50 μg of total protein were used for all the described assays and data were normalized on the sample protein content [6].

**ATP and AMP intracellular content evaluation**

For each assay, 50 μg of total protein were used. ATP was assayed spectrophotometrically following NADP reduction at 340 nm. The assay medium contained: 100 mM Tris-HCl (pH 8.0), 0.2 mM NADP, 5 mM MgCl_2_, and 50 mM glucose. Samples were analyzed before and after the addition of 3 μg of purified hexokinase plus glucose-6-phosphate dehydrogenase.

AMP was assayed spectrophotometrically following NADH oxidation at 340 nm. Reaction medium contained: 100 mM Tris-HCl (pH 8.0), 5 mM MgCl_2_, 0.2 mM ATP, 10 mM phosphoenolpyruvate, 0.15 mM NADH, 10 IU adenylate kinase, 25 IU pyruvate kinase, and 15 IU of lactate dehydrogenase. The ratio between the intracellular concentrations of ATP and AMP (ATP/AMP) represents a marker of cellular energy status [7].

**Oxygen consumption assay**

Oxygen consumption rate (OCR) was measured with an amperometric electrode (Unisense Microrespiration, Unisense A/S, Denmark) in a closed chamber at 25 °C. For each experiment, 2x10^5^ cells were resuspended in phosphate buffer saline (PBS) and permeabilized with 0.03 mg/ml digitonin for 1 min. To stimulate the pathways composed of complexes I, III, and IV, or II, III, and IV, 10 mM pyruvate plus 5 mM malate or 20 mM succinate were used, respectively [6]. To test the cellular affinity for glucose, glutamine, and fatty acids as respiratory substrates, cells were maintained in the growth medium and 4 µM BPTES (a glutaminase inhibitor [8]), 4 µM Etomoxir (a fatty acid oxidation inhibitor [9]), and 4 µM UK5099 (a mitochondrial pyruvate carrier inhibitor [10]) were added. Data were expressed as nmol O­/min/10^6^ cells.

**FoF1 ATP-synthase activity assay**

The F_o_F_1_ ATP-synthase (ATP Synthase) activity was evaluated by incubating 2x10^5^ cells at 25 °C for 10 min in a medium containing: 50 mM Tris-HCl (pH 7.4), 50 mM KCl, 1 mM EGTA, 2 mM MgCl_2_, 0.6 mM ouabain, 0.25 mM di(adenosine)-5-Penta-phosphate (an adenylate kinase inhibitor), and 25 μg/mL ampicillin (0.1 mL final volume). 10 mM pyruvate plus 5 mM malate or 20 mM succinate were employed to stimulate complexes I, III, and IV or complexes II, III, and IV pathways, respectively [7, 11]. As for OCR evaluation, 4 µM BPTES, 4 µM Etomoxir, and 4 µM UK5099 were used for the cellular energy substrate affinity evaluation. In this case, cells are suspended in the growth medium diluted 1:1 with the solution described above. In each case, ATP synthesis was induced by adding 0.1 mM ADP. The reaction was monitored every 30 sec for 2 min with a luminometer (GloMax® 20/20 Luminometer, Promega Italia), using the luciferin/luciferase chemiluminescent method (luciferin/luciferase ATP bioluminescence assay kit CLS II, Roche, Basel, Switzerland). ATP standard solutions in a concentration range between 10^-8^ and 10^-5^ M were used for calibration. Data were expressed as nmol ATP/min/10^6^ cells [6].

**P/O ratio**

The P/O value is calculated as the ratio between aerobic ATP synthesis and oxygen consumption and represents an OxPhos efficiency marker. Efficient mitochondria display a P/O value of 2.5 when stimulated with pyruvate plus malate or 1.5 in the presence of succinate. When the P/O value is around the reference values, all the oxygen consumed is devoted to energy production, and the OXPHOS metabolism is defined as coupled. By contrast, a lower P/O ratio suggests that part of the oxygen is not employed for energy production but may contribute to the reactive oxygen species (ROS) formation, and OXPHOS is defined as uncoupled [12].

**Enzymatic antioxidant defenses and lipid peroxidation damage assays**

For each assay 50 μg of total protein were used. Glucose 6-phosphate dehydrogenase (G6PD) activity was assayed spectrophotometrically at 340 nm following NADP reduction. The assay mix contained: Tris-HCl (pH 7.4), 0.5 mM NADP, and 10 mM glucose-6-phosphate [13].

Glutathione reductase (GR) activity was assayed spectrophotometrically at 340 nm, following the NADPH oxidation. The assay medium contained: 100 mM Tris-HCl (pH 7.4), 1 mM EDTA, 5 mM GSSG, and 0.2 mM NADPH [11].

Catalase activity was assayed spectrophotometrically following the H2O2 decomposition at 240 nm. The assay mix contained: 50 mM phosphate buffer (pH 7.0), and 5 mM H_2_O_2_ [6].

Malondialdehyde (MDA) concentration was assessed to evaluate lipid peroxidation damages, using the thiobarbituric acid reactive substances (TBARS) assay. This test is based on the reaction of thiobarbituric acid (TBA) with MDA, a breakdown product of lipid peroxides. The TBARS solution contained 26 mM thiobarbituric acid and 15% trichloroacetic acid (TCA) in 0.25 N HCl. To evaluate MDA concentration, 50 μg of total protein dissolved in 300 μl of Milli-Q water were added with 600 μl of TBARS solution. The mix was incubated at 95 °C for 60 min. The sample was centrifuged at 14,000 rpm for 2 min, and the supernatant was then analyzed spectrophotometrically at 532 nm [14].

**Mitochondrial mass and superoxide production measurement**

RS-PDXs-derived and primary CLL cells were thawed or freshly cultured with RPMI 10% FCS for 3 hours. Cells were washed with PBS and for mitochondrial mass evaluation, incubated with 10 nM MitoTracker green (ThermoFisher Scientific) for 15 min at 37 °C, while for superoxide production measurement, incubated with 5 μM MitoSOX Red (ThermoFisher Scientific) for 20 min at 37 °C. After treatment, cells were washed and analyzed by flow cytometry.

**Histochemical detection of enzymatic activities**

The enzyme histochemical procedures were based on the protocols described by Miller and colleagues [15]. Briefly, tumor masses were harvested, cut in 100 mm^2^ pieces, and snap-frozen in O.C.T. compound (Sigma Aldrich, Milan, Italy) and store at -80 °C. Tissue blocks were then trimmed in a cryostat chamber, cut in 6 μm thickness, mounted on Superfrost Plus micro slides (Thermo Fisher Scientific, Waltham, USA), and stored at -80 °C until use. For the enzymatic activity staining, enzyme specific buffers were prepared as follows. For G6PD and LDH assay, the buffer was freshly prepared containing 0.1 M Tris-Maleate buffer pH 7.5, 0.45 mM methoxyphenanzine methosulfate, 5 mM sodium azide and 5 mM nitroblue tetrazolium chloride (NBT, pre-dissolved in 70% dimethylformamide and heated to 60 °C). The assay media also contained 15 mM G6P, 0.8 mM NADP and 4 mM MgCl_2_ for the analysis of G6PD activity; 150 mM sodium lactate and 3 mM NAD^+^ for the analysis of LDH activity. Negative control reactions were performed in the absence of substrate. Before performing the experiment, tissue sections were defrosted for five minutes in a moist chamber. Assay medium containing the enzyme-specific substrate and coenzymes was applied to the slide to cover the whole tissue section. Enzyme reactions were carried out protected from light at room temperature for 15 minutes. Slides were washed twice in 60 °C PBS for one minute and once in 4 °C PBS for three minutes to remove the incubation media and stop the reaction. Tissue sections were directly dried and mounted in Mowiol 4-88. Stained sections were acquired with Axio Lab A1 microscope (Zeiss, Oberkochen, DE) at 4X and 40X magnifications. Accumulation of NBT aggregates was identified and scored in a semi-quantitative manner as the percentage of NBT precipitation positive area.

**qRT-PCR**

RNA was extracted using the RNeasy Plus Mini kit (Qiagen, Hilden, DE) and retrotranscribed to cDNA using the High-Capacity cDNA Reverse Transcription kit (Thermo Fisher Scientific, Waltham, USA). qRT-PCR was performed using iTaq Universal Probes Supermix (Bio-Rad, Hercules, USA) and the following probes: *CS* (Hs02574374_s1), *OGDH* (Hs01081865_m1), *LDHA* (Hs00855332_g1), *GLS* (Hs01014020_m1), *GLUD1* (Hs03989560_s1), *HADH* (Hs00997450_m1), *CAT* (Hs00156308_m1), *G6PD* (Hs00166169_m1), *FASN* (Hs01005622_m1), *MECR* (Hs00211238_m1), and the housekeeping gene *B2M* (Hs00984230_m1), all from Life Technologies. Plates were run in the CFX384 Real-Time System (Bio-Rad, Hercules, USA) using the following PCR conditions: one cycle at 95 °C for 1 min, followed by 40 cycles at 95 °C for 15 s and 60 °C for 1 min. Reactions were done in triplicate from the same cDNA (technical replicates). The difference (ΔCT) between the target gene cycle threshold (CT) and *B2M* CT was calculated and data were plotted as gene copy numbers.

**Western blot**

Western blot was performed as previously described [16]. Briefly, approximately 2x10^6^ cells were lysed and the total protein amount was quantified with the Bradford method using Bio-Rad Protein Assay Dye Reagent Concentrate (Bio-Rad, Hercules, USA), according to the manufacturer’s protocol. Proteins were resolved by SDS-PAGE, using 4-20% pre-casted gradient Mini-PROTEAN TGX Stain-Free Gels and transferred onto a 0.2 μm nitrocellulose Trans-Blot Turbo Transfer membrane (all from Bio-Rad). Membranes were blocked with 5% non-fat milk in Tris-buffered saline with Tween (TBS-T) for 1 hour and then incubated overnight (O.N.) at 4 °C with primary antibodies in 5% BSA or 5% non-fat milk in TBS-T. The following primary antibodies were used: Citrate Synthase D7V8B Rabbit mAb (#14309, Cell Signaling Technology, Danvers, USA), α-KGDH E1W8H Rabbit mAb (#26865, Cell Signaling Technology), LDHA C4B5 Rabbit mAb (#3582 Cell Signaling Technology), HADH antibody (ab154088, Abcam, Cambridge, UK), Glutaminase-1/GLS1 E4T9Q Rabbit mAb (#49363, Cell Signaling Technology), Catalase EPR20198 (ab209211, Abcam), Glucose 6 Phosphate Dehydrogenase EPR6291 (ab124738, Abcam), solute carrier family 2 member 1 (SLC1A1; GLUT1; #BK12939, Cell Signaling Technology), solute carrier family 2 member 5 (SLC2A5; GLUT5; #BK71407, Cell Signaling Technology), solute carrier family 2 member 4 (SLC2A4; GLUT4; #07-1404, Sigma Aldrich), solute carrier family 7 member 5 (SLC7A5; LAT1; #BK5347, Cell Signaling Technology), solute carrier family 1 member 5 (SLC1A5; ASCT2; #BK5345, Cell Signaling Technology), solute carrier family 38 member 5 (SLC38A5; SNAT5; #Ab72717, Abcam) and β-Actin C4 HRP (sc-47778, Santa Cruz Biotechnology). After incubation with appropriate secondary antibodies, images were acquired using ChemiDoc Touch Imaging System (Bio-Rad). The optical density of the bands was determined with the Image Lab 3.0 software (Bio-Rad), and protein levels were normalized using the β-actin signal.

**Ex-vivo treatment of RS cells and apoptosis evaluation**

Peripheral blood mononuclear cells (PBMCs) from healthy donors, U-RT1, and RS-PDXs cells were cultured for 48 hours in the following conditioned media i) RPMI, ii) RPMI + 10 mM glucose, iii) RPMI + 2 mM glutamine, iv) RPMI + 10 mM glucose + 2 mM glutamine, v) RPMI + 10 mM glucose + 2 mM glutamine + 10% FCS, in the absence or presence of 4 μM of the glutaminase GLS1 inhibitor BPTES (BPT), 4 μM of the carnitine palmitoyltransferase-1 CPT-1 inhibitor Etomoxir (ETO), and/or 4 μM of the mitochondrial pyruvate carrier inhibitor UK5099 (UK5). After treatment, cells were collected, and viability assessed by Annexin V-APC Apoptosis Kit (ThermoFisher Scientific) following manufacturer’s instructions. Samples were analyzed by flow cytometry using FACS Celesta (BD Biosciences).

**Proliferation assay**

RS-PDXs-derived and CLL cells were freshly used for proliferation assay. Approximately 2x10^5^ cells were washed with PBS and fix/perm with Ethanol 70% for 1 hour at -20 °C. Cells were washed twice with PBS 1% BSA and stained with Ki-67 antibody (Miltenyi Biotec, Bergisch Gladbach, DE) for 20 min at room temperature, before FACS analysis.

**Untargeted high-resolution mass spectrometry (HRMS) metabolomic analyses**

3x10^6^ cells pellet was extracted adding 550 µL of a mixture CHCl_3_:MeOH 2:1, maintained on ice for 40 minutes and 400 µL of H_2_O added. After centrifugation (10 minutes at 14,000xg at 4 °C), 450 µL supernatant were taken to dryness and stored at -80 °C until analyzed. A 100 µL of H_2_O:MeOH 50:50 and 5 microliter of IS kit (MSK-QC-KIT) were then added to the samples and 10 µl were injected in the Vanquish UHPLC system (ThermoFisher Scientific). Quality control (QC) samples were prepared by pooling together supernatants obtained from all the samples.

LC-HRMS analysis was carried out using a Vanquish Horizon UHPLC system coupled to a Q-Exactive Plus Hybrid Quadrupole-Orbitrap Mass Spectrometer (ThermoFisher Scientific). The liquid chromatographic separation was carried out using two different chromatographic conditions: reversed phase and HILIC. Reversed phase gradient separation chromatography was carried out using an ACQUITY BEH C18 (2.1 mm X 100 mm, i.d. 1.7 µm, Waters, Milan, Italy) with mobile phase A consisting of 0.1% formic acid in water, and mobile phase B of 0.1 % formic acid in acetonitrile. The percentage of solvent B started at 1% for 0.1 min, reached 100% in 15 min and was kept for 5 min at a flow rate of 250 μL/min, then the column was reconditioned at 1% B for 5 min for a total run time of 25 min. The column temperature was maintained at 40 °C. HILIC phase gradient separation chromatography was carried out using ACQUITY BEH Amide (2.1 mm X 150 mm, i.d. 1.7 µm, Waters, Milan, Italy) with mobile phase A consisting of 5 mM ammonium formate, pH 3, in water and mobile phase B of acetonitrile. The percentage of solvent B started at 10% for 0.1 min, reached 70% in 15 min and was kept for 5 min at a flow rate of 200 μL/min, then the column was reconditioned at 1% B for 9 min for a total run time of 27 min. The column temperature was maintained at 25 °C. Ionization was obtained using a heated electrospray source probe both in positive and negative mode with spray voltages at 3.9 kV and 3.7 kV, respectively. The capillary temperature was set at 300 °C. Nitrogen sheath and auxiliary gas were set at a flow rate of 35 and 5 arbitrary units respectively. The analyses were performed using two different acquisition modes with an m/z range of 70–1200. The first acquisition mode, used for compound profiling purposes, was a full MS scan mode with the following parameters: resolution of 70,000, auto gain control target <1×10^5^ and maximum injection time of 100 ms. The other acquisition mode, used for the identification of unknown compounds, was a full MS scan acquisition followed by data-dependent MS2 (DDA) with a resolution of 17,500, an auto gain control target of 1×10^5^, a maximum injection time of 65 ms, a loop count of top 5 peaks and an isolation window of m/z 1.2. All MS2 spectra of the compounds were acquired at 3 collision energies: 20, 40, and 80 eV. The MS1 mass range was divided into 10 mass ranges with a width of 50 m/z each on which the data dependent analysis was carried out. DDA was performed with a priority fragmentation for the m/z of our Accurate Mass Retention Time (AMRT) library [17]. Samples were randomized in the analytical sequence to avoid bias due to instrument drift.

The data were processed with MS-DIAL ver.4.8 software [18] for deconvolution, peak picking, alignment, and compound identification. The four master area tables obtained by MS-DIAL, generated by combining the two different chromatographic methods and the two different MS polarities were exported as a .txt file. The dataset, composed of four different cellular models and QCs, was imported, and statistically analyzed in Perseus (https://maxquant.net/perseus/). The expression data was log2 transformed and the QC group was removed. Each feature was, therefore, filtered by row with a valid value of at least 70% for each group. The missing data were replaced and normalization by subtraction of the median was used to normalize the intensity values. Indeed, for the annotations of in silico compounds, starting from ionic features with associated MS2 events, MS-FINDER ver.3.26 was used [19]. The MS-FINDER annotation matrix was merged with the quantitative matrix by employing the matching row by name Perseus option and the Alignment ID was used as the unique classifier. Only the MS-FINDER Structure Rank score>5 was used. iPath3.0 was used for the visualization and analysis of cellular pathways [20].

**References**

1. Beekman R, Chapaprieta V, Russinol N, Vilarrasa-Blasi R, Verdaguer-Dot N, Martens JHA, Duran-Ferrer M, Kulis M, Serra F, Javierre BM *et al*: **The reference epigenome and regulatory chromatin landscape of chronic lymphocytic leukemia**. *Nat Med* 2018, **24**(6):868-880.

2. Nadeu F, Royo R, Massoni-Badosa R, Playa-Albinyana H, Garcia-Torre B, Duran-Ferrer M, Dawson KJ, Kulis M, Diaz-Navarro A, Villamor N *et al*: **Detection of early seeding of Richter transformation in chronic lymphocytic leukemia**. *Nat Med* 2022, **28**(8):1662-1671.

3. Damiani C, Rovida L, Maspero D, Sala I, Rosato L, Di Filippo M, Pescini D, Graudenzi A, Antoniotti M, Mauri G: **MaREA4Galaxy: Metabolic reaction enrichment analysis and visualization of RNA-seq data within Galaxy**. *Comput Struct Biotechnol J* 2020, **18**:993-999.

4. Bradford MM: **A rapid and sensitive method for the quantitation of microgram quantities of protein utilizing the principle of protein-dye binding**. *Anal Biochem* 1976, **72**:248-254.

5. Cappelli E, Cuccarolo P, Stroppiana G, Miano M, Bottega R, Cossu V, Degan P, Ravera S: **Defects in mitochondrial energetic function compels Fanconi Anaemia cells to glycolytic metabolism**. *Biochim Biophys Acta Mol Basis Dis* 2017, **1863**(6):1214-1221.

6. Cappelli E, Degan P, Bruno S, Pierri F, Miano M, Raggi F, Farruggia P, Mecucci C, Crescenzi B, Naim V *et al*: **The passage from bone marrow niche to bloodstream triggers the metabolic impairment in Fanconi Anemia mononuclear cells**. *Redox Biol* 2020, **36**:101618.

7. Cappelli E, Bertola N, Bruno S, Degan P, Regis S, Corsolini F, Banelli B, Dufour C, Ravera S: **A Multidrug Approach to Modulate the Mitochondrial Metabolism Impairment and Relative Oxidative Stress in Fanconi Anemia Complementation Group A**. *Metabolites* 2021, **12**(1).

8. Vacanti NM, Divakaruni AS, Green CR, Parker SJ, Henry RR, Ciaraldi TP, Murphy AN, Metallo CM: **Regulation of substrate utilization by the mitochondrial pyruvate carrier**. *Mol Cell* 2014, **56**(3):425-435.

9. O'Connor RS, Guo L, Ghassemi S, Snyder NW, Worth AJ, Weng L, Kam Y, Philipson B, Trefely S, Nunez-Cruz S *et al*: **The CPT1a inhibitor, etomoxir induces severe oxidative stress at commonly used concentrations**. *Sci Rep* 2018, **8**(1):6289.

10. Zhong Y, Li X, Yu D, Li X, Li Y, Long Y, Yuan Y, Ji Z, Zhang M, Wen JG *et al*: **Application of mitochondrial pyruvate carrier blocker UK5099 creates metabolic reprogram and greater stem-like properties in LnCap prostate cancer cells in vitro**. *Oncotarget* 2015, **6**(35):37758-37769.

11. Ravera S, Bertola N, Pasquale C, Bruno S, Benedicenti S, Ferrando S, Zekiy A, Arany P, Amaroli A: **808-nm Photobiomodulation Affects the Viability of a Head and Neck Squamous Carcinoma Cellular Model, Acting on Energy Metabolism and Oxidative Stress Production**. *Biomedicines* 2021, **9**(11).

12. Hinkle PC: **P/O ratios of mitochondrial oxidative phosphorylation**. *Biochim Biophys Acta* 2005, **1706**(1-2):1-11.

13. Salani B, Ravera S, Amaro A, Salis A, Passalacqua M, Millo E, Damonte G, Marini C, Pfeffer U, Sambuceti G *et al*: **IGF1 regulates PKM2 function through Akt phosphorylation**. *Cell Cycle* 2015, **14**(10):1559-1567.

14. Ravera S, Dufour C, Cesaro S, Bottega R, Faleschini M, Cuccarolo P, Corsolini F, Usai C, Columbaro M, Cipolli M *et al*: **Evaluation of energy metabolism and calcium homeostasis in cells affected by Shwachman-Diamond syndrome**. *Sci Rep* 2016, **6**:25441.

15. Miller A, Nagy C, Knapp B, Laengle J, Ponweiser E, Groeger M, Starkl P, Bergmann M, Wagner O, Haschemi A: **Exploring Metabolic Configurations of Single Cells within Complex Tissue Microenvironments**. *Cell Metab* 2017, **26**(5):788-800 e786.

16. Iannello A, Vitale N, Coma S, Arruga F, Chadburn A, Di Napoli A, Laudanna C, Allan JN, Furman RR, Pachter JA *et al*: **Synergistic efficacy of the dual PI3K-delta/gamma inhibitor duvelisib with the Bcl-2 inhibitor venetoclax in Richter syndrome PDX models**. *Blood* 2021, **137**(24):3378-3389.

17. Lavarello C, Barco S, Bartolucci M, Panfoli I, Magi E, Tripodi G, Petretto A, Cangemi G: **Development of an Accurate Mass Retention Time Database for Untargeted Metabolomic Analysis and Its Application to Plasma and Urine Pediatric Samples**. *Molecules* 2021, **26**(14).

18. Tsugawa H, Cajka T, Kind T, Ma Y, Higgins B, Ikeda K, Kanazawa M, VanderGheynst J, Fiehn O, Arita M: **MS-DIAL: data-independent MS/MS deconvolution for comprehensive metabolome analysis**. *Nat Methods* 2015, **12**(6):523-526.

19. Lai Z, Tsugawa H, Wohlgemuth G, Mehta S, Mueller M, Zheng Y, Ogiwara A, Meissen J, Showalter M, Takeuchi K *et al*: **Identifying metabolites by integrating metabolome databases with mass spectrometry cheminformatics**. *Nat Methods* 2018, **15**(1):53-56.

20. Darzi Y, Letunic I, Bork P, Yamada T: **iPath3.0: interactive pathways explorer v3**. *Nucleic Acids Res* 2018, **46**(W1):W510-W513.

**Supplemental Tables**

| **NAME** | **SIZE** | **ES** | **NES** | **NOM p-val** | **FDR q-val** |
| --- | --- | --- | --- | --- | --- |
| HALLMARK_GLYCOLYSIS | 198 | 0.59532845 | 1.949716 | 0 | 0.017873095 |
| HALLMARK_DNA_REPAIR | 149 | 0.6838874 | 1.913516 | 0 | 0.012825416 |
| HALLMARK_OXIDATIVE_PHOSPHORYLATION | 200 | 0.7556699 | 1.9029701 | 0 | 0.008550277 |
| HALLMARK_FATTY_ACID_METABOLISM | 156 | 0.5916824 | 1.8904163 | 0 | 0.008295395 |
| HALLMARK_REACTIVE_OXYGEN_SPECIES_PATHWAY | 49 | 0.7017072 | 1.8341453 | 0 | 0.009899238 |
| HALLMARK_PEROXISOME | 104 | 0.5792989 | 1.8303467 | 0 | 0.008575426 |
| HALLMARK_ADIPOGENESIS | 198 | 0.5518055 | 1.826882 | 0 | 0.007350365 |
| HALLMARK_MTORC1_SIGNALING | 197 | 0.7171959 | 1.8040173 | 0 | 0.007609987 |
| HALLMARK_MYC_TARGETS_V1 | 195 | 0.7958221 | 1.803312 | 0 | 0.006764433 |
| HALLMARK_MYC_TARGETS_V2 | 57 | 0.81766135 | 1.7304893 | 0 | 0.013450854 |
| HALLMARK_ESTROGEN_RESPONSE_LATE | 197 | 0.44692856 | 1.6977057 | 0 | 0.01690773 |
| HALLMARK_G2M_CHECKPOINT | 195 | 0.73854494 | 1.6974517 | 0 | 0.015498751 |
| HALLMARK_UNFOLDED_PROTEIN_RESPONSE | 109 | 0.5917283 | 1.6935748 | 0.012376238 | 0.014886813 |
| HALLMARK_UV_RESPONSE_UP | 156 | 0.51176727 | 1.6920085 | 0.008522728 | 0.013823468 |
| HALLMARK_CHOLESTEROL_HOMEOSTASIS | 73 | 0.5359562 | 1.6842188 | 0.006329114 | 0.01381133 |
| HALLMARK_E2F_TARGETS | 198 | 0.79253036 | 1.5972221 | 0 | 0.031257316 |
| HALLMARK_HYPOXIA | 197 | 0.41833436 | 1.5687119 | 0.030100334 | 0.03670306 |
| HALLMARK_SPERMATOGENESIS | 133 | 0.42056435 | 1.5261298 | 0.017994858 | 0.048688896 |
| HALLMARK_NOTCH_SIGNALING | 32 | 0.49140576 | 1.5212704 | 0.026946107 | 0.047693387 |
| HALLMARK_MITOTIC_SPINDLE | 198 | 0.48627523 | 1.5145221 | 0.046035804 | 0.04744645 |
| HALLMARK_ANDROGEN_RESPONSE | 99 | 0.44271642 | 1.5122076 | 0.01861702 | 0.04602037 |
| HALLMARK_PI3K_AKT_MTOR_SIGNALING | 105 | 0.45024827 | 1.5095055 | 0.039534885 | 0.044678476 |
| HALLMARK_P53_PATHWAY | 196 | 0.41328624 | 1.438805 | 0.074927956 | 0.06986805 |
| HALLMARK_XENOBIOTIC_METABOLISM | 197 | 0.36394322 | 1.3290119 | 0.0882353 | 0.13613479 |
| HALLMARK_PROTEIN_SECRETION | 95 | 0.44860706 | 1.3012692 | 0.1541756 | 0.1505865 |
| HALLMARK_ANGIOGENESIS | 36 | 0.376444 | 1.2957383 | 0.14769231 | 0.14988263 |
| HALLMARK_BILE_ACID_METABOLISM | 112 | 0.354914 | 1.2161436 | 0.19711539 | 0.21727875 |
| HALLMARK_IL2_STAT5_SIGNALING | 198 | 0.32431456 | 1.2027439 | 0.20216607 | 0.22214895 |
| HALLMARK_ESTROGEN_RESPONSE_EARLY | 198 | 0.2925562 | 1.1943206 | 0.17218544 | 0.2229555 |
| HALLMARK_APICAL_JUNCTION | 199 | 0.27651808 | 1.0735947 | 0.33636364 | 0.34841734 |
| HALLMARK_APOPTOSIS | 160 | 0.2988603 | 1.062825 | 0.3483871 | 0.34780288 |
| HALLMARK_ALLOGRAFT_REJECTION | 196 | 0.3079335 | 1.0599397 | 0.3525836 | 0.3403296 |
| HALLMARK_PANCREAS_BETA_CELLS | 40 | 0.30738932 | 1.0497229 | 0.3742072 | 0.34347358 |
| HALLMARK_EPITHELIAL_MESENCHYMAL_TRANSITION | 197 | 0.24079648 | 1.0043501 | 0.41735536 | 0.3922881 |
| HALLMARK_MYOGENESIS | 198 | 0.2171096 | 0.90941024 | 0.61172163 | 0.5246981 |
| HALLMARK_HEME_METABOLISM | 194 | 0.25889966 | 0.9058719 | 0.5802139 | 0.51804805 |
| HALLMARK_INTERFERON_GAMMA_RESPONSE | 197 | 0.27431715 | 0.8864421 | 0.5646067 | 0.5389211 |
| HALLMARK_COMPLEMENT | 200 | 0.24183942 | 0.8515056 | 0.6779141 | 0.59026414 |
| HALLMARK_UV_RESPONSE_DN | 141 | 0.22110133 | 0.7821787 | 0.8205805 | 0.70702606 |
| HALLMARK_HEDGEHOG_SIGNALING | 36 | 0.23129852 | 0.75021136 | 0.84 | 0.75212646 |
| HALLMARK_WNT_BETA_CATENIN_SIGNALING | 42 | 0.23217948 | 0.72638613 | 0.87401575 | 0.77469724 |
| HALLMARK_INTERFERON_ALPHA_RESPONSE | 95 | 0.25353864 | 0.7207861 | 0.7764128 | 0.7664985 |
| HALLMARK_TNFA_SIGNALING_VIA_NFKB | 198 | 0.20569992 | 0.59454143 | 0.8746518 | 0.9157792 |
| **Supplemental Table 1**. GSEA pathways in primary RS from whom RS-PDX models were established. In green are highlighted the statistically significant pathways. | | | | | |

| **NAME** | **SIZE** | **ES** | **NES** | **NOM p-val** | **FDR q-val** |
| --- | --- | --- | --- | --- | --- |
| HALLMARK_OXIDATIVE_PHOSPHORYLATION | 181 | 0.5627313 | 1.8037465 | 0.007067138 | 0.15731716 |
| HALLMARK_ADIPOGENESIS | 157 | 0.40844163 | 1.5614679 | 0.008196721 | 0.21596818 |
| HALLMARK_FATTY_ACID_METABOLISM | 124 | 0.38536456 | 1.5601159 | 0.050473187 | 0.17427936 |
| HALLMARK_REACTIVE_OXYGEN_SPECIES_PATHWAY | 44 | 0.4696955 | 1.5507771 | 0.0569395 | 0.15611771 |
| HALLMARK_DNA_REPAIR | 135 | 0.41263995 | 1.5469737 | 0.05755396 | 0.1374708 |
| HALLMARK_MYC_TARGETS_V1 | 190 | 0.5456667 | 1.5428269 | 0.1557377 | 0.12339712 |
| HALLMARK_CHOLESTEROL_HOMEOSTASIS | 61 | 0.45341238 | 1.536937 | 0.05533597 | 0.11294598 |
| HALLMARK_MYC_TARGETS_V2 | 57 | 0.6461856 | 1.5193722 | 0.03827751 | 0.113432534 |
| HALLMARK_ESTROGEN_RESPONSE_LATE | 126 | 0.3556442 | 1.5123613 | 0.03539823 | 0.10750705 |
| HALLMARK_G2M_CHECKPOINT | 183 | 0.5153148 | 1.5085447 | 0.10243902 | 0.10147724 |
| HALLMARK_UV_RESPONSE_UP | 117 | 0.3726106 | 1.4243715 | 0.07364341 | 0.14180176 |
| HALLMARK_E2F_TARGETS | 191 | 0.5759125 | 1.4171034 | 0.07514451 | 0.13968715 |
| HALLMARK_MTORC1_SIGNALING | 186 | 0.526182 | 1.6112148 | 0.016064256 | 0.29950458 |
| HALLMARK_GLYCOLYSIS | 147 | 0.40081173 | 1.5766864 | 0.032 | 0.25845465 |
| HALLMARK_UNFOLDED_PROTEIN_RESPONSE | 102 | 0.33072284 | 1.2135081 | 0.25981873 | 0.34313005 |
| HALLMARK_NOTCH_SIGNALING | 27 | 0.30123645 | 1.1437631 | 0.25 | 0.42097354 |
| HALLMARK_PEROXISOME | 86 | 0.27343032 | 1.1408733 | 0.25872093 | 0.40088284 |
| HALLMARK_PI3K_AKT_MTOR_SIGNALING | 88 | 0.2802042 | 1.1169094 | 0.29131654 | 0.41621923 |
| HALLMARK_ALLOGRAFT_REJECTION | 140 | 0.26612136 | 1.114367 | 0.3099631 | 0.39878574 |
| HALLMARK_SPERMATOGENESIS | 63 | 0.2715488 | 1.062029 | 0.37777779 | 0.46231708 |
| HALLMARK_ANDROGEN_RESPONSE | 83 | 0.262586 | 1.0619467 | 0.32890365 | 0.44030198 |
| HALLMARK_HYPOXIA | 140 | 0.23057924 | 1.0419844 | 0.3950178 | 0.45118243 |
| HALLMARK_INFLAMMATORY_RESPONSE | 122 | 0.25830755 | 1.0332679 | 0.371134 | 0.4457321 |
| HALLMARK_XENOBIOTIC_METABOLISM | 118 | 0.22336392 | 1.0232422 | 0.39130434 | 0.44125625 |
| HALLMARK_P53_PATHWAY | 167 | 0.22534959 | 0.99677074 | 0.43984962 | 0.4624745 |
| HALLMARK_ESTROGEN_RESPONSE_EARLY | 130 | 0.20354845 | 0.9821859 | 0.46638656 | 0.46760517 |
| HALLMARK_IL2_STAT5_SIGNALING | 157 | 0.2170751 | 0.96948504 | 0.48863637 | 0.47175875 |
| HALLMARK_TNFA_SIGNALING_VIA_NFKB | 166 | 0.25958133 | 0.872374 | 0.56832296 | 0.63757044 |
| HALLMARK_EPITHELIAL_MESENCHYMAL_TRANSITION | 95 | 0.16985703 | 0.76032925 | 0.8455285 | 0.82894605 |
| HALLMARK_INTERFERON_GAMMA_RESPONSE | 168 | 0.17654857 | 0.7011699 | 0.8111888 | 0.91238004 |
| HALLMARK_COMPLEMENT | 137 | 0.16636239 | 0.6897526 | 0.9067164 | 0.8997554 |
| HALLMARK_BILE_ACID_METABOLISM | 73 | 0.16424492 | 0.6628215 | 0.9320652 | 0.902419 |
| HALLMARK_PROTEIN_SECRETION | 88 | 0.18406227 | 0.6566736 | 0.8449612 | 0.882326 |
| HALLMARK_IL6_JAK_STAT3_SIGNALING | 58 | 0.14242151 | 0.5539003 | 0.9775281 | 0.9487194 |
| **Supplemental Table 2**. GSEA pathways in RS-PDX models. In green are highlighted the statistically significant pathways. | | | | | |

| SAMPLE ID | PHENOTYPE | BINET STAGE | *IGHV* | *TP53* | CYTOGENETICS | CD38 |
| --- | --- | --- | --- | --- | --- | --- |
| CLL1 | Indolent | A | M | mut | Trisomy 12 | Neg |
| CLL2 | Indolent | A | UM | wt | Normal | Neg |
| CLL3 | Indolent | A | M | wt | Del11q22; del13q14 | Neg |
| CLL4 | Indolent | B | UM | wt | Normal | Neg |
| CLL5 | Aggressive | C | UM | mut | Del11q22; del13q14 | Pos |
| CLL6 | Aggressive | C | UM | mut | Del13q14 | Pos |
| CLL7 | Aggressive | C | UM | wt | Del17p13 | Pos |
| CLL8 | Aggressive | C | UM | wt | Del17p13; del13q14;  trisomy 12 | Pos |
|  | | | | | | |
| Supplemental Table 3. Main clinical, genetic/cytogenetic, and molecular features of CLL patients included in enzymatic activity analyses. M: mutated; UM: unmutated; mut: mutated; wt: wild type; del: deletion; Neg: negative (<20%); Pos: positive (≥20%) | | | | | | |

| SAMPLE TYPE | PHENOTYPE | RAI STAGE | *IGHV* | *TP53* | CYTOGENETICS | CD38 |
| --- | --- | --- | --- | --- | --- | --- |
| CLL9 | Indolent | 0 | M | wt | NA | Neg |
| CLL10 | Indolent | 0 | M | NA | NA | Neg |
| CLL11 | Indolent | 0 | M | wt | NA | Neg |
| CLL12 | Indolent | 0 | M | NA | Del13q14 | Neg |
| CLL13 | Indolent | 1 | M | NA | NA | Pos |
| CLL14 | Indolent | 1 | M | wt | Normal | Pos |
| CLL15 | Indolent | 0 | M | wt | Normal | Neg |
| CLL16 | Indolent | 1/2 | M | wt | Del13q14.3 | Neg |
| CLL17 | Indolent | 0 | M | wt | Del13q14.3 | Neg |
| CLL18 | Indolent | 0 | M | wt | Del13q14.3 | Neg |
| CLL19 | Indolent | 0 | UM | wt | Normal | Neg |
| CLL20 | Aggressive | 2 | UM | mut | Trisomy12 | Pos |
| CLL21 | Aggressive | 4 | UM | wt | Del13q14.3; del*TP53* | Neg |
| CLL22 | Aggressive | 3 | UM | mut | Del13q14.3 | Pos |
| CLL23 | Aggressive | 2 | UM | NA | Del*ATM*; del17p; del13q14.3 | Pos |
| CLL24 | Aggressive | 2 | UM | NA | Trisomy12 | Pos |
| CLL25 | Aggressive | 2 | UM | wt | Trisomy12 | Pos |
| CLL26 | Aggressive | 2 | UM | NA | Del13q14.3; del11q22.3; trisomy12 | Pos |
| CLL27 | Aggressive | 0 | UM | wt | Trisomy12; *IGHV* gene rearrangements | Pos |
| CLL28 | Aggressive | 1 | UM | wt | Trisomy12 | Pos |
| CLL29 | Aggressive | 0 | UM | mut | Del17p | Neg |
| CLL30 | Aggressive | 1 | UM | wt | Trisomy12; del13q14.3 | Pos |
|  |  |  |  |  |  |  |
| Supplemental Table 4. Main clinical, genetic/cytogenetic, and molecular features of CLL patients included in the analysis of enzymes expression. NA: not available; M: mutated; UM: unmutated; mut: mutated; wt: wild type; del: deletion; Neg: negative (<20%); Pos: positive (≥20%) | | | | | | |

**Supplemental Figure legends**

**Supplemental Fig. S1. Principal component analysis (PCA) in U-RT1 cell line and RS compared to CLL. (a-b)** Over-representation analysis (ORA) of up-regulated genes and their corresponding Gene Ontology (GO) terms in primary RS (**a**) and RS-PDXs (**b**) versus CLL samples. Oxidative phosphorylation, glycolysis, reactive oxygen species, and fatty acid metabolism terms are highlighted in red. **(c-g)** PCA of PDX-corresponding primary samples (n=4), PDXs (n=13), and primary CLL samples (n=349). CLL data were obtained from publicly available datasets (GSE92626, GSE66117, GSE176141, GSE119103, EGAD00001004046, and EGAD00001000258). Only the genes belonging to the oxidative phosphorylation **(c)**, reactive oxygen species **(d)**, glycolysis **(e)**, fatty acid **(f)**, and glutamate-glutamine metabolism **(g)** were considered as input for the analyses, respectively. (**h**) Genes related to metabolic terms (1442 genes) from PathCards were represented as log_10_ TPM (transcript per million reads) in primary and their corresponding PDXs.

**Supplemental Fig. S2. Transcript and protein expression levels of selected metabolic enzymes. (a)** Histochemical detection of the enzymatic activity of the lactate dehydrogenase (LDH; representative images) in tumor masses sections of RS-PDX models (magnification X4; insets X40). Box plot represents the percentage of LDH activity positive area in 5 independent experiments. **(b)** Expression, by quantitative reverse transcriptase polymerase chain reaction (qRT-PCR) analyses, in CLL and RS cells of selected genes coding for metabolic enzymes. mRNA expression is normalized over b*-2-microglobulin* (*B2M*). **(c)** Western blot panels showing protein expression of selected metabolic enzymes. Box plots represent band intensities obtained from different samples (run in separate experiments). Band intensities were quantified using Image Lab software and normalized on b-actin, used as loading control. **(d)** Bar plots reporting differences in band intensities (from western blot data) between indolent and aggressive CLL patients.

Data are reported as mean ± SEM. Statistical analysis was performed using 1-way analysis of variance (ANOVA); *P .05, **P .01, ***P .001, ****P .0001; red asterisks summarize statistical significance between RS and CLL, while black ones show statistical significance among RS-PDX models.

**Supplemental Fig. S3. Glucose uptake in RS-PDX models and CLL patients. (a)** Graph showing the 2-NBDG uptake kinetics, plotted as mean fluorescence intensity (MFI) measured by flow cytometry after 1, 3, 5, 10, 15, and 30 minutes of incubation. **(b)** Box plot showing the expression levels, reported as transcript per kilobase million (TPM), of selected glucose transporters (GLUT5/*SLC2A5* and GLUT4/*SLC2A4*) in CLL patients and RS-PDX models. **(c)** Box plot showing the expression levels, reported as TPM, of selected glutamine transporters (Sodium-dependent amino acids transporter: *SLC1A5* and *SLC38A5*) in CLL patients and RS-PDX models. (**d-e**) Western blot analysis (**d**) of the expression, at the protein level, of the main glucose and glutamine transporters and quantification (**e**) of the corresponding bands, expressed as a ratio over actin, used as a loading control. Data are reported as mean ± SEM. Statistical analysis was performed using 1-way analysis of variance (ANOVA); *P .05, **P .01, ***P .001, ****P .0001.

**Supplemental Fig. S4. OXPHOS activity, expression levels and activity of antioxidant enzymes, and lipid peroxidation levels. (a-b),** Energetic status and mitochondrial activity of CLL and RS-PDX-derived cells represented as aerobic ATP synthesis (molecules per million cells; **a**) and oxygen consumption rate (OCR; **b**). Data reported in **A-B** were obtained using 20 mM succinate as a respiratory substrate. **(c)** Analysis, by flow cytometry using the MitoTRACKER Green mitochondrial staining, of mitochondrial content in CLL and RS cells. Data are shown as a box plot reporting the mean fluorescence intensity (MFI). **(d)** Box plots showing the activity of catalase (CAT) and glutathione reductase (GRX), both involved in the antioxidant response. **(e)** Quantitative reverse transcriptase polymerase chain reaction analyses of *CAT* and *GLRX*. Messenger RNA expression is normalized over *b-2-microglobulin* (*B2M*). **(f)** Evaluation of Malondialdehyde (MDA) content as a marker of lipid peroxidation.

**Supplemental Fig. S5. Expression levels of enzymes involved in anabolic pathways.** **(a)** Quantitative reverse transcriptase polymerase chain reaction analyses of *G6PD, FASN,* and *MECR*. Messenger RNA expression is normalized over *b-2-microglobulin* (*B2M*). **(b)** Western blot panel showing expression of G6PD. Box plot represents band intensities in 5 independent experiments. Band intensities were measured using Image Lab and normalized on b-actin. **(c)** Box plots showing G6PD expression in aggressive versus indolent CLL patients.

**Supplemental Fig. S6. ATP synthesis and OCR in RS-PDXs following metabolic inhibitors exposure. (a-b)** ATP production **(a)** and O_2_ consumption **(b)** by RS cells in the absence or presence of selective metabolic inhibitors (BPTES/BPT: glutaminase inhibitor; Etomoxir/ETO: carnitine palmitoyltransferase 1 inhibitor; UK5099/UK5: mitochondrial pyruvate carrier inhibitor) plotted as nmol per minute (min) per million cells. **(c)** Impact of BPT, ETO, and UK5 on ATP synthesis and OCR comparing CLL and RS cells.

**Supplemental Fig. S7. Metabolic dependencies of the U-RT1 cell line. (a)** ATP production and **(b)** O_2_ consumption of the U-RT1 cell line in the presence or absence of selective metabolic inhibitors (BPTES/BPT: glutaminase inhibitor; Etomoxir/ETO: carnitine palmitoyltransferase1 inhibitor; UK5099/UK5: mitochondrial pyruvate carrier inhibitor). Data are shown as the quantity of ATP produced **(a)** or oxygen consumption **(b)** in a minute from 10^6^ cells (left panel) and as a percentage of residual activity compared to the untreated condition (right panel). **(c)** Oxygen consumption rate of U-RT1 cells in the presence of sequential injections of selective metabolic inhibitors (BPTES; Etomoxir; UK5099). **(d)** Apoptotic assay measuring the substrate dependencies of RS cells (glucose: Glu; glutamine: Gln; fatty acid: fetal bovine serum FBS which is enriched in fatty acids). Bar plots report the percentage of viable cells after 48 hours of culture in complete or substrate-deprived media.

Data are reported as mean ± SEM. Statistical analysis was performed using 1-way or 2-way analysis of variance (ANOVA); *P .05, **P .01, ***P .001, ****P .0001.

**Supplemental Fig. S8.** **Impact of PI3K and NF-kB targeting on RS cells metabolism. (a)** Basal respiration of RS cells following PI3K inhibitor (Duvelisib; 5µM) or NF-kB inhibitor (SC75741; 5µM) exposure for 24h. Data are shown as oxygen consumption rate (OCR) measured with Seahorse XF-24 Extracellular Flux Analyzer using the Mitostress kit. **(b)**Glycolytic capacity of RS cells following PI3K inhibitor (Duvelisib; 5µM) or NF-kB inhibitor (SC75741; 5µM) exposure for 24h. Data are shown as the Extracellular acidification rate (ECAR) measured with Seahorse XF-24 Extracellular Flux Analyzer using the Glycolysis stress kit. **(c)**Apoptotic assay showing the percentage of viable RS cells NF-kB inhibitor (SC75741; 5µM) exposure for 24 hours. **(d)** Apoptotic assay showing the percentage of viable U-RT1 in the presence of PI3K inhibitor (Duvelisib; 5µM) for 24h. **(c-d)** Data reported as mean ± SEM. Statistical analysis was performed using 1-way or 2-way analysis of variance (ANOVA); *P .05, **P .01, ***P .001, ****P .0001. **c-d,** Data reported as mean ± SEM. Statistical analysis was performed using t-test; *P .05, **P .01, ***P .001, ****P .0001.

**
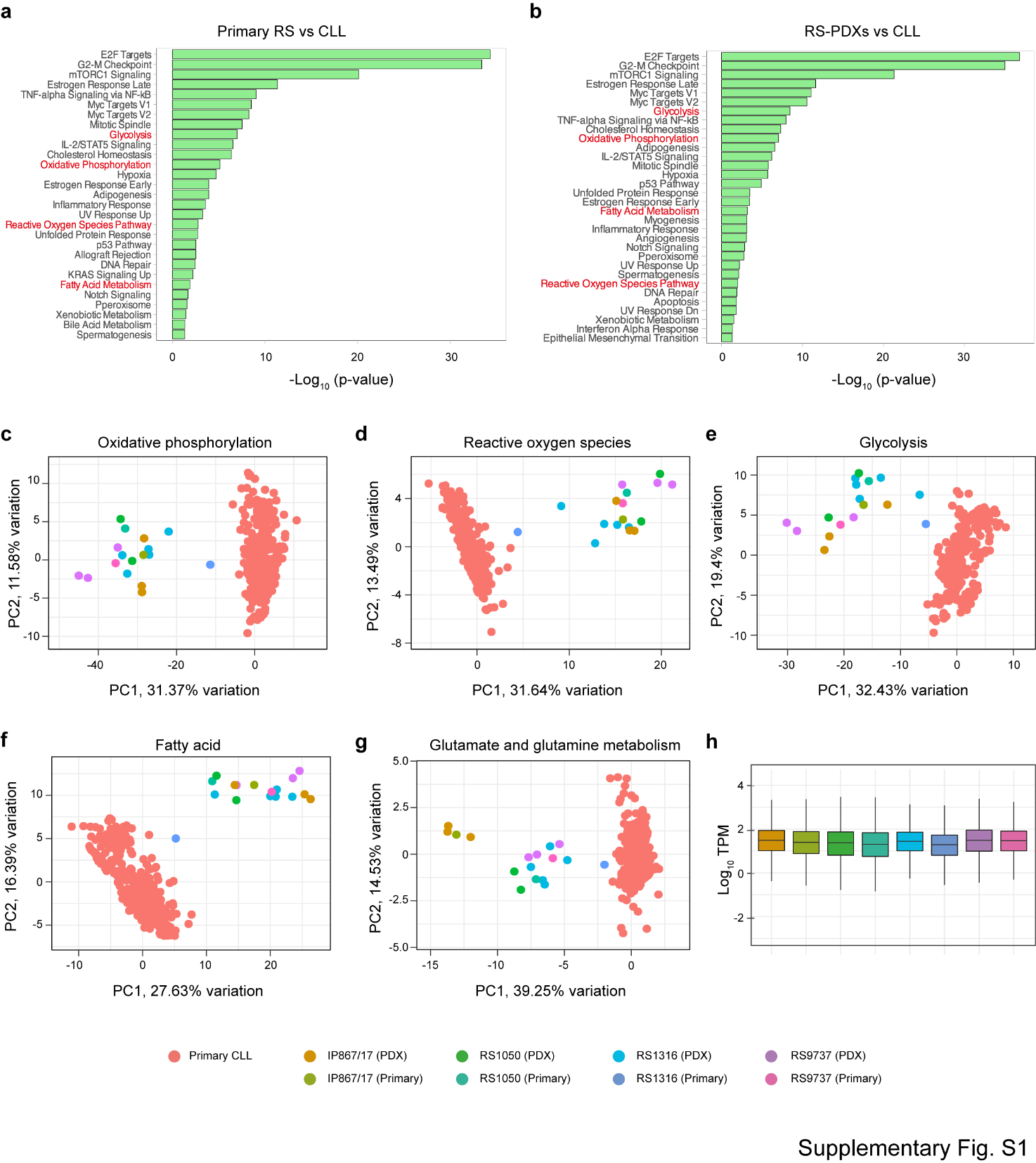
**

**
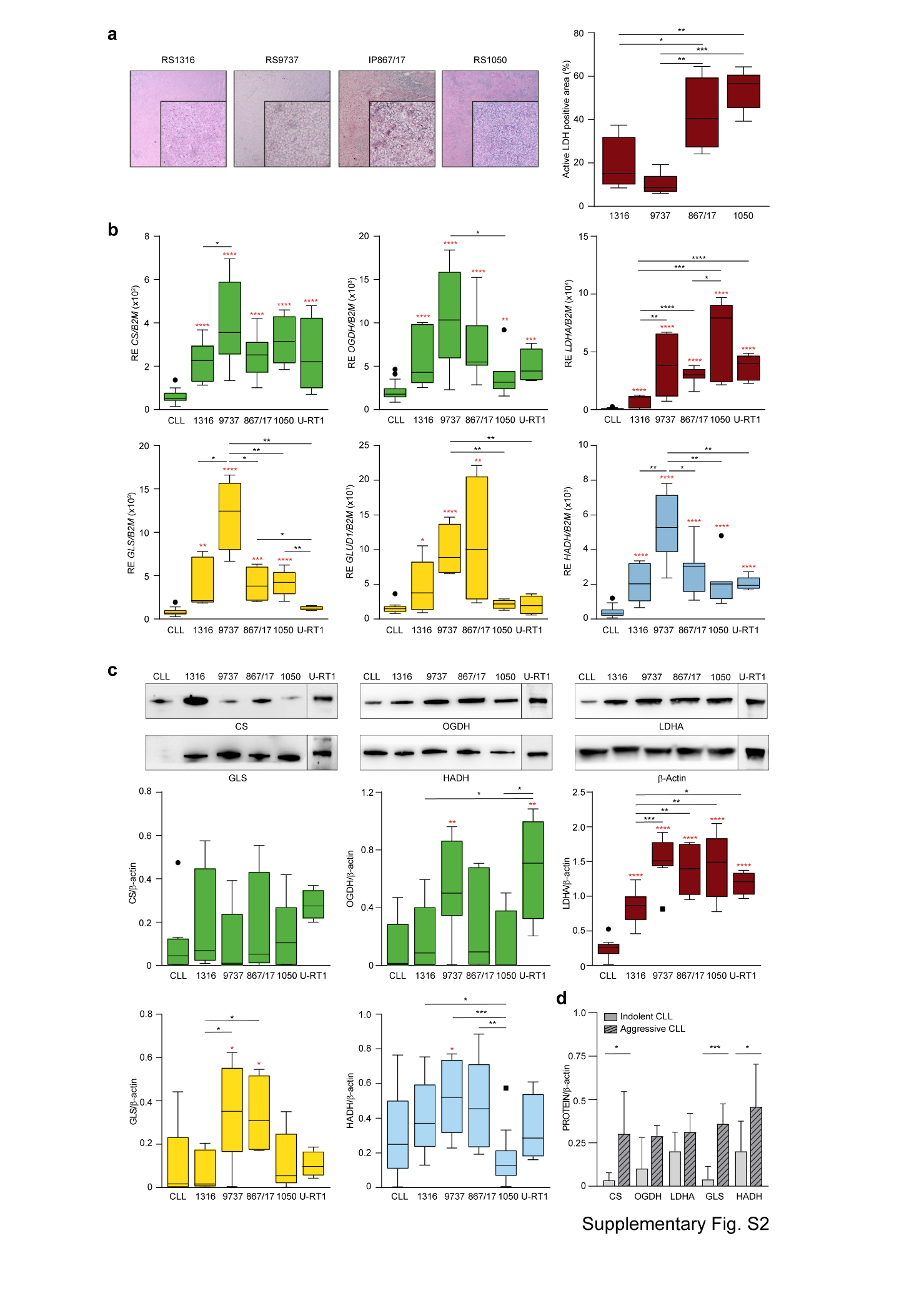
**

**
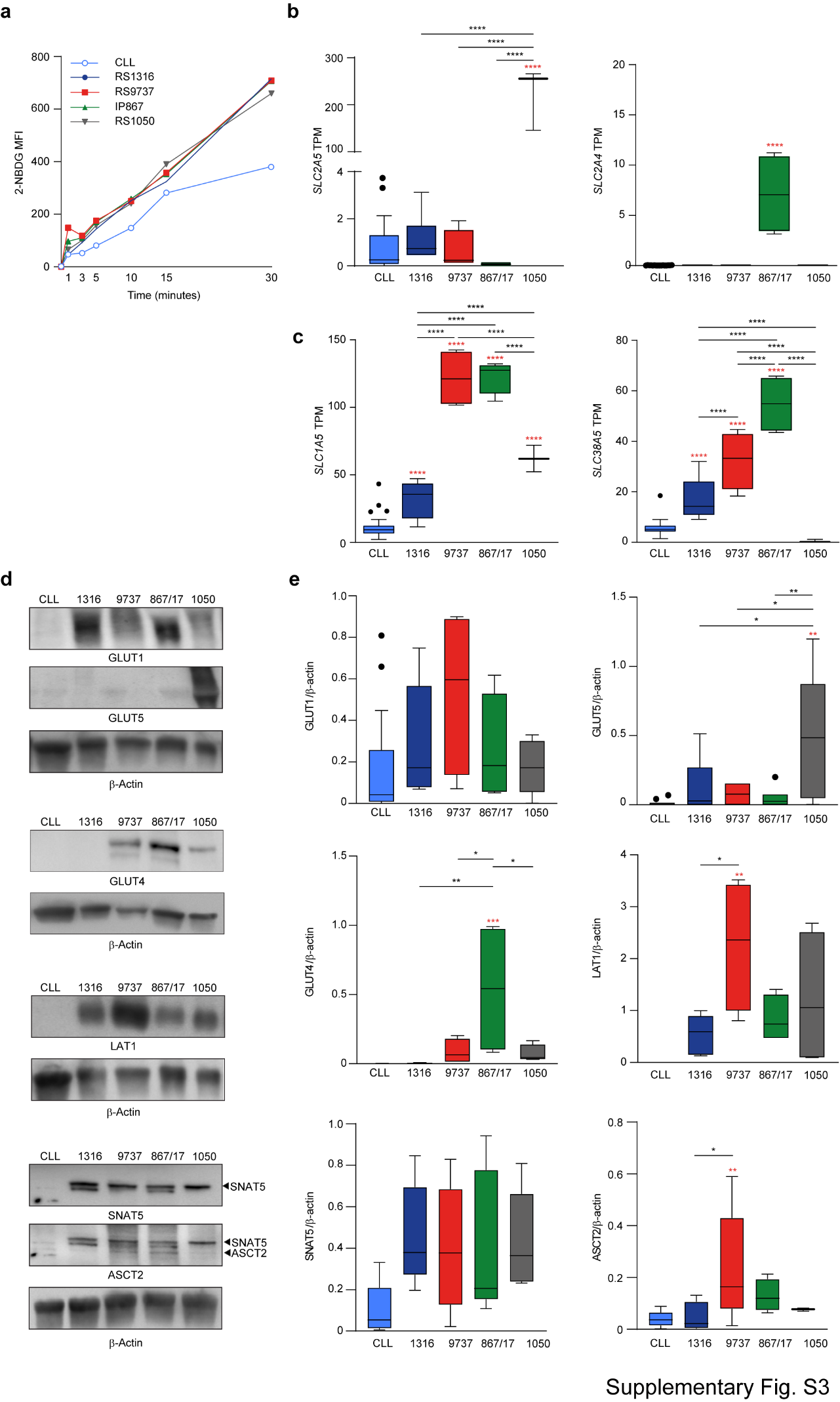
**

**
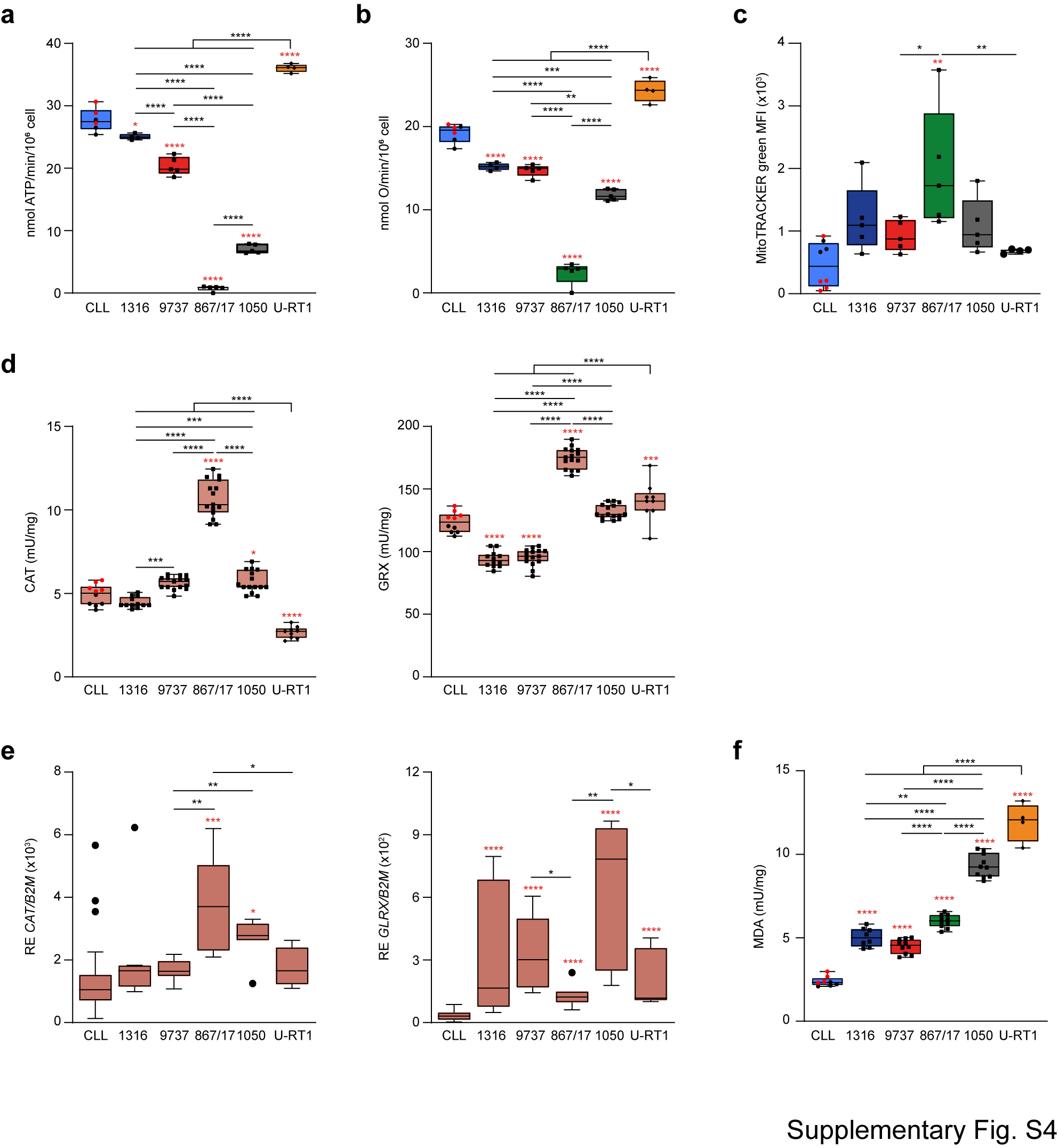
**

**
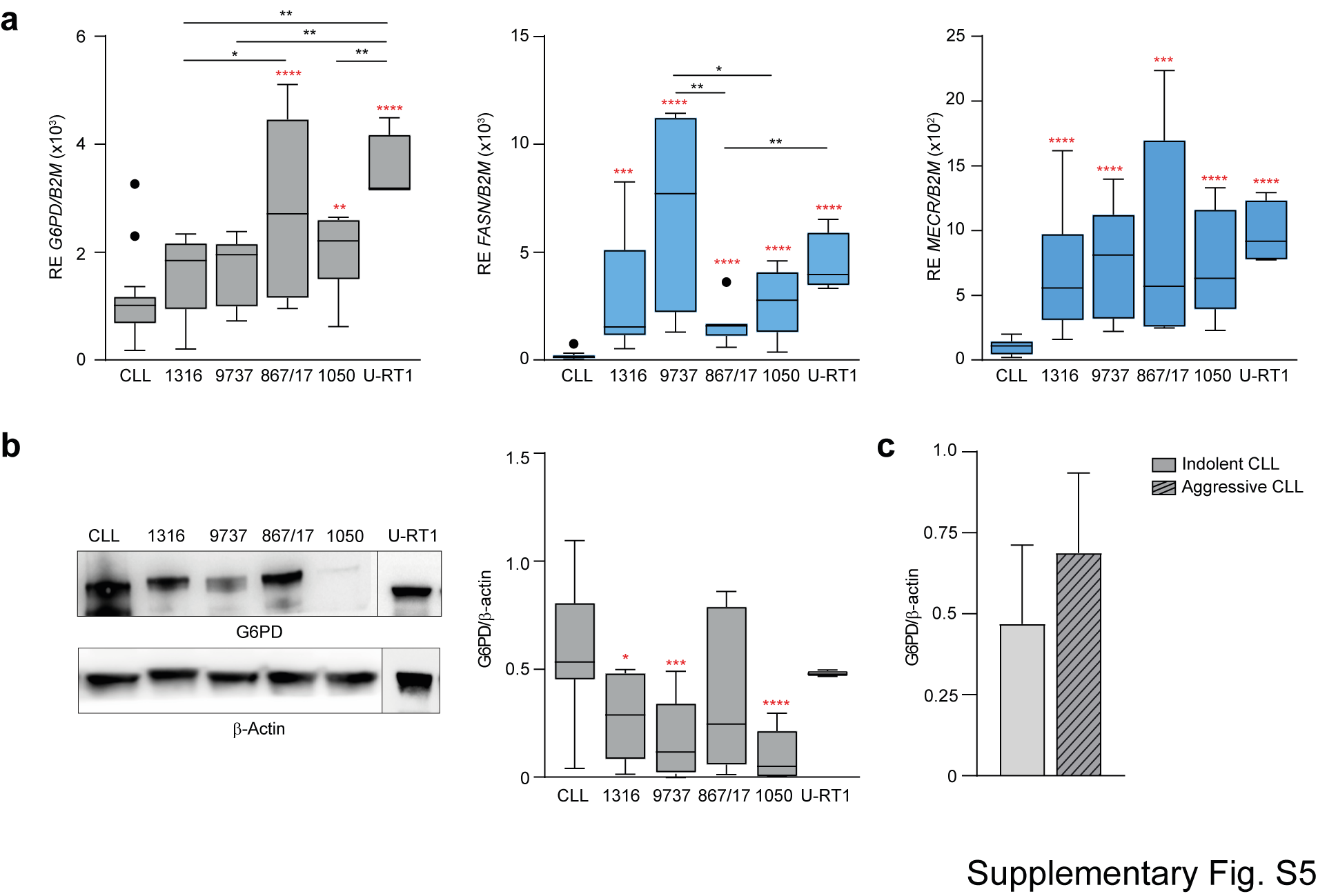
**

**
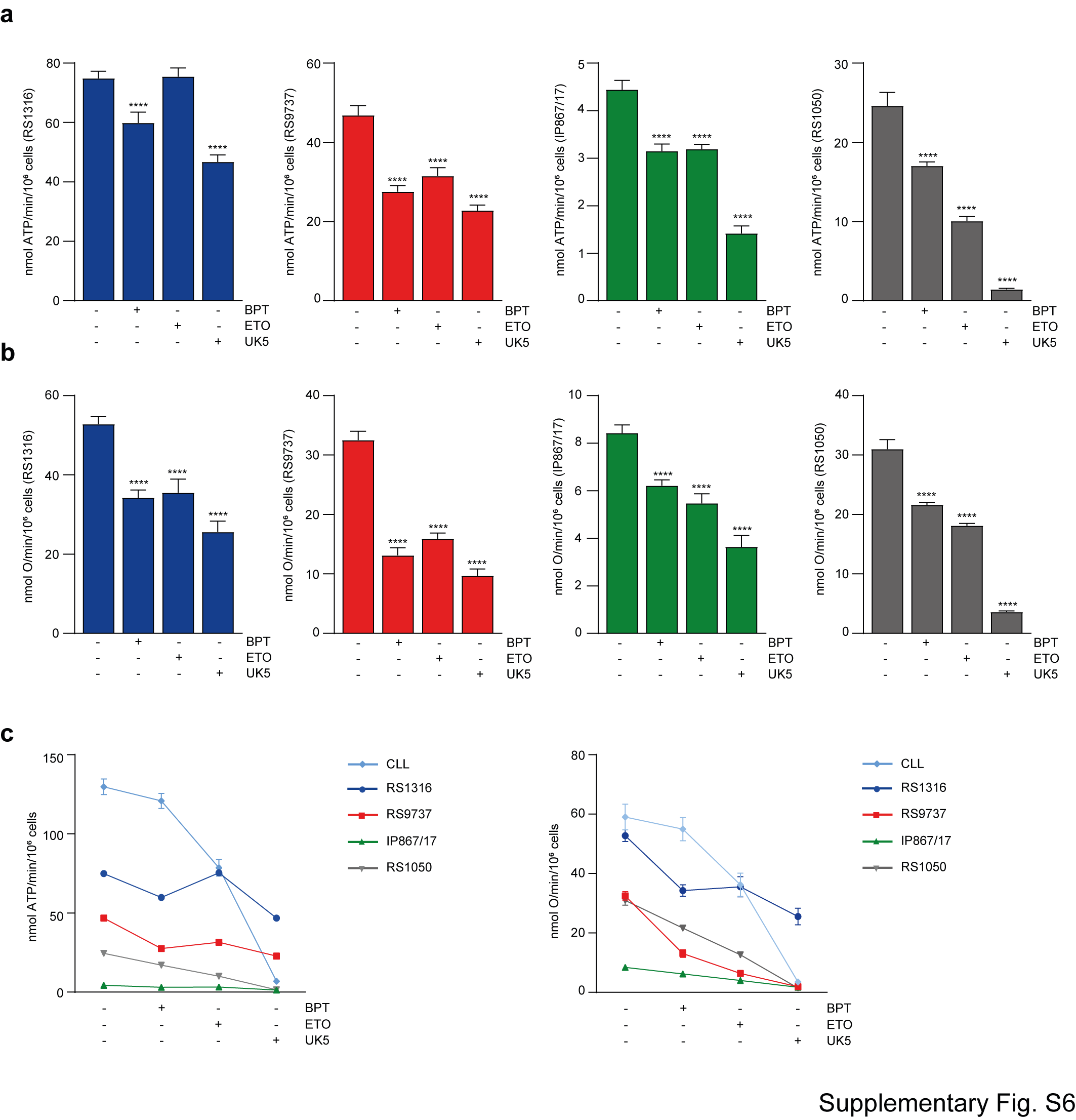
**

**
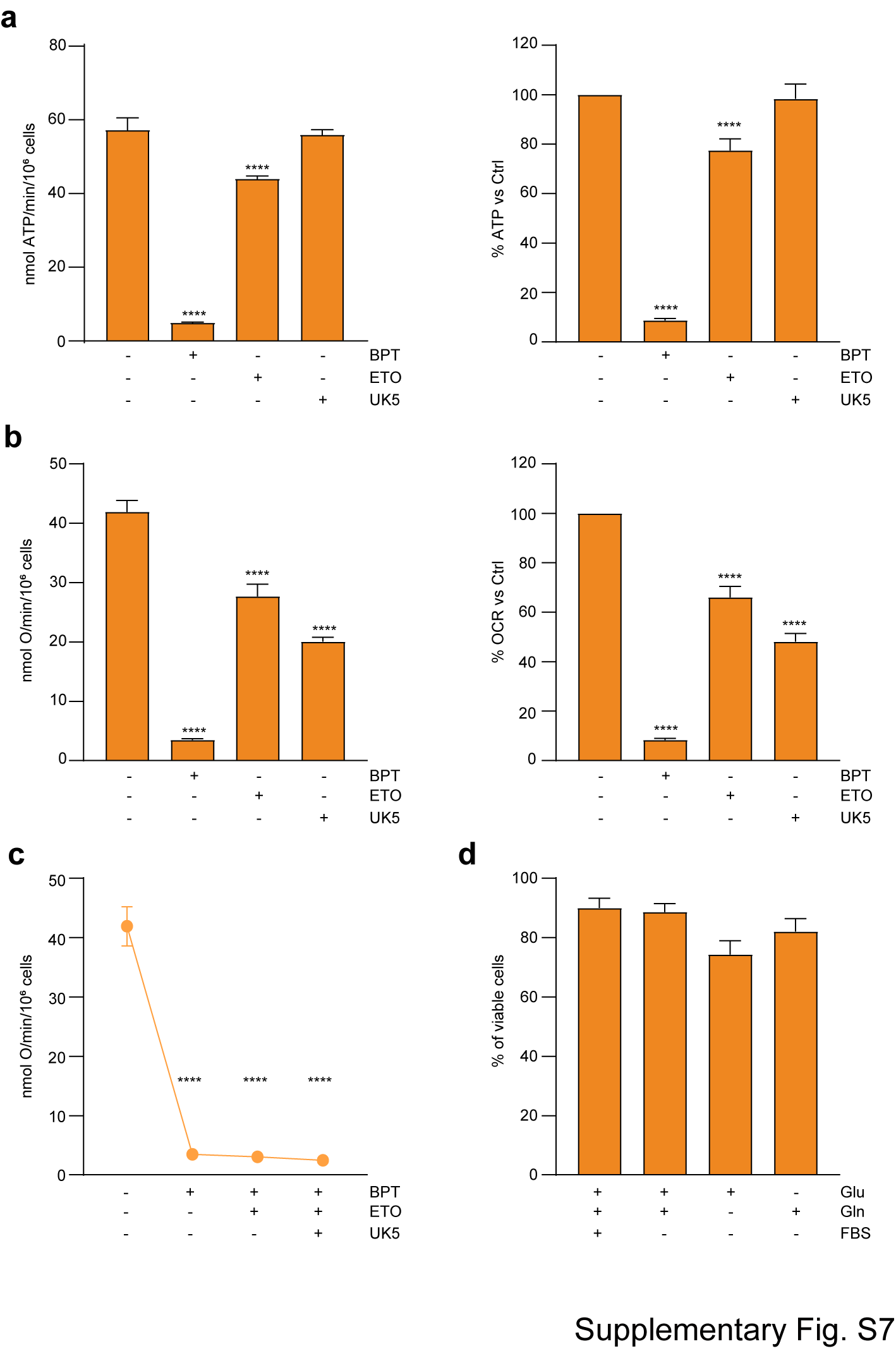

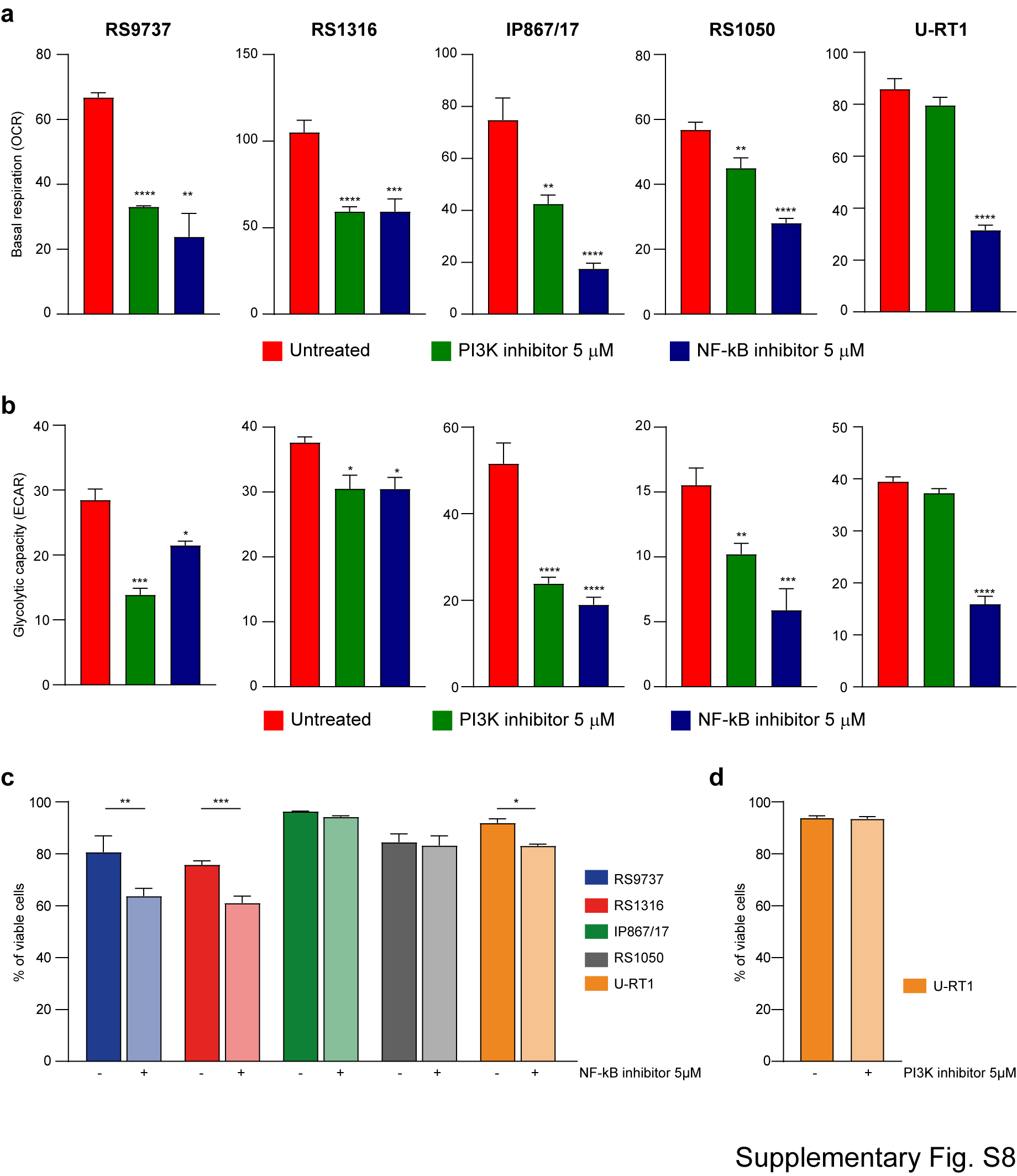
**
